# Targeting signaling pathways in prostate cancer: mechanisms and clinical trials

**DOI:** 10.1038/s41392-022-01042-7

**Published:** 2022-06-24

**Authors:** Yundong He, Weidong Xu, Yu-Tian Xiao, Haojie Huang, Di Gu, Shancheng Ren

**Affiliations:** 1 Shanghai Key Laboratory of Regulatory Biology, School of Life Sciences, East China Normal University, Shanghai, China; 2 Department of Urology, Shanghai Changzheng Hospital, Shanghai, China; 3 Department of Urology, Shanghai Changhai Hospital, Shanghai, China; 4 Department of Urology, Mayo Clinic College of Medicine and Science, Rochester, MN USA; 5 Department of Urology, The First Affiliated Hospital of Guangzhou Medical University, Guangzhou, Guangdong China

**Keywords:** Cancer therapy, Urological cancer

## Abstract

Prostate cancer (PCa) affects millions of men globally. Due to advances in understanding genomic landscapes and biological functions, the treatment of PCa continues to improve. Recently, various new classes of agents, which include next-generation androgen receptor (AR) signaling inhibitors (abiraterone, enzalutamide, apalutamide, and darolutamide), bone-targeting agents (radium-223 chloride, zoledronic acid), and poly(ADP-ribose) polymerase (PARP) inhibitors (olaparib, rucaparib, and talazoparib) have been developed to treat PCa. Agents targeting other signaling pathways, including cyclin-dependent kinase (CDK)4/6, Ak strain transforming (AKT), wingless-type protein (WNT), and epigenetic marks, have successively entered clinical trials. Furthermore, prostate-specific membrane antigen (PSMA) targeting agents such as ^177^Lu-PSMA-617 are promising theranostics that could improve both diagnostic accuracy and therapeutic efficacy. Advanced clinical studies with immune checkpoint inhibitors (ICIs) have shown limited benefits in PCa, whereas subgroups of PCa with mismatch repair (MMR) or CDK12 inactivation may benefit from ICIs treatment. In this review, we summarized the targeted agents of PCa in clinical trials and their underlying mechanisms, and further discussed their limitations and future directions.

## Introduction

Prostate cancer (PCa) is the second most common cancer, and it is the fifth leading cause of cancer-related death among men.^1^ The incidence rates of PCa are 37.5 per 100,000 in developed countries and 11.3 per 100,000 in developing countries, while mortality rates are 8.1 per 100,000 in developed countries and 5.9 per 100,000 in developing countries.^1^ Approximately 10 million men are presently diagnosed with PCa. PCa causes more than 400,000 deaths annually worldwide, and by 2040, the mortality rate is expected to reach more than 800,000 deaths annually.^1–4^ Prostate-specific antigen (PSA) testing and digital rectal examinations (DRE) facilitate the diagnosis of PCa in most men at early stages of the disease.^5,6^ Androgen receptor (AR) signaling plays an essential role in PCa initiation and disease progression,^7^ and androgen deprivation therapy (ADT) has been a backbone of treatment for patients with advanced disease.^8,9^ Generally, localized PCa is managed by deferred treatment or active local therapy (such as radical prostatectomy or radiation therapy) with or without ADT. For metastatic PCa, ADT with gonadotropin-releasing hormone (GnRH) antagonists/agonists followed by treatment with docetaxel plus prednisolone and continued ADT after disease progression has become the standard treatment.^10^ However, patient responses to ADT vary, and most patients eventually develop castration-resistant prostate cancer (CRPC).^11^ In the past decade, significant progress has been made in the treatment of CRPC; this progress has been aided by the approval of effective AR-targeting agents, including abiraterone, enzalutamide, apalutamide, and darolutamide.^12–16^ The agents newly approved in the 21st century for PCa treatment and diagnosis are summarized in Table 1.

**Table 1:** Theranostic agents approved for PCa in the 21st century

International non-proprietary name (INN)	Brand name	Pharmacotherapeutic group	EMA approval date	FDA approval date	China NMPA approval date
Zoledronic acid	Zometa	Bone-targeting therapy	Mar 2001	Feb 2002	Dec 2018
Degarelix	Firmagon	Endocrine therapy	Feb 2009	Dec 2008	July 2019
Sipuleucel-T	Provenge	Immunotherapy	Sept 2013	Apr 2010	/
Cabazitaxel	Jevtana	Antineoplastic agents	Mar 2011	Jun 2010	/
Denosumab	Prolia/Xgeva	Bone-targeting therapy	July 2011	Nov 2010	May 2019
Abiraterone	Zytiga	Endocrine therapy	Sept 2011	Apr 2011	Dec 2019
Enzalutamide	Xtandi	Endocrine therapy	Jun 2013	Aug 2012	Nov 2019
Radium-223 dichloride	Xofigo	Therapeutic radiopharmaceuticals	Nov 2013	May 2013	Aug 2020
Fluciclovine (18F)	Axumin	Diagnostic radiopharmaceuticals	May 2017	May 2016	/
Pembrolizumab	Keytruda	Immunotherapy	/	May 2017	/
Padeliporfin	Tookad	Antineoplastic agents	Sept 2017	/	/
Darolutamide	Nubeqa	Endocrine therapy	Mar 2020	Jul 2019	Feb 2021
Rucaparib	Rubraca	Antineoplastic agents	/	May 2020	/
Olaparib	Lynparza	Antineoplastic agents	Nov 2020	May 2020	Jun 2021
Relugolix	Orgovyx	Endocrine therapy	Mar 2022	Dec 2020	/
Piflufolastat F 18	Pylarify	Diagnostic radiopharmaceuticals	/	May 2021	/
Dostarlimab-gxly	Jemperli	Immunotherapy	/	Aug 2021	/
^177^Lu-PSMA-617	Pluvicto	Therapeutic radiopharmaceuticals	/	Mar 2022	/
^68^Ga-PSMA-11	Locametz	Diagnostic radiopharmaceuticals	/	Mar 2022	/

Bone metastasis is a major concern in patients with CRPC. Successful therapeutic strategies for the treatment of bone metastases include radium 223, bisphosphonates, and receptor activator of nuclear factor kappa-B ligand (RANKL) inhibitor denosumab.^17–22^ Treatments targeting genomic alterations in DNA repair pathways have been increasingly validated in clinical settings. Poly(ADP-ribose) polymerase (PARP) inhibitors, including olaparib, rucaparib, and talazoparib, are being evaluated in phase 2/3 trials for metastatic castration-resistant prostate cancer (mCRPC).^23–27^ Moreover, early clinical studies on agents that target immune checkpoints, such as cytotoxic T-lymphocyte associated protein 4 (CTLA4), programmed cell death protein 1 (PD1) or programmed death-ligand 1 (PD-L1) have been evaluated in clinics.^28–32^ Prostate-specific membrane antigen (PSMA) is highly expressed in PCa cell membranes.^33,34^ Thus, PSMA-targeting small molecules or antibodies labeled with radionuclides or cytostatic agents have been evaluated in several clinical studies.^35–45^ Moreover, multifarious cell growth and survival pathways, including phosphatidylinositol-3-kinase (PI3K)/Ak strain transforming (AKT)/mechanistic target of rapamycin (mTOR), interact with AR signaling, and are involved in PCa progression. Single-agent treatment with PI3K/AKT/mTOR specific inhibitors or combination approaches with AR signaling inhibitors have been investigated in clinical studies.^46–52^ Alterations of epigenetic modifications, such as histone methylation and acetylation, as well as DNA methylation, are ubiquitous in PCa.^53,54^ Therefore, compounds concerning epigenetic targets, such as lysine methyltransferase (KMT), histone lysine demethylase (KDM), histone acetyltransferase (HAT), bromodomain and extra-terminal (BET), histone deacetylase (HDAC) or DNA methyltransferase (DNMT), have entered clinical trials.^54–62^ Agents targeting other PCa-related signaling pathways, including cyclin-dependent kinase (CDK)4/6, p53, wingless-type protein (WNT) signaling, vascular endothelial growth factor (VEGF), endothelin A receptor (ETAR), fibroblast growth factor receptor (FGFR), epidermal growth factor receptor (EGFR), receptor tyrosine kinases (RTKs), transforming growth factor beta (TGFβ), proto-oncogene tyrosine-protein kinase Src (SRC), and mitogen-activated protein kinase kinase (MEK), have also entered clinical trials.^63–74^ Alternative splicing affects genes (such as *FGFR*, *ERG*, *VEGFA*, and *AR*) that are clearly linked to the etiology of PCa; therefore, developing novel targeted therapies that modulate alternative splicing for the treatment of PCa are warranted.^75^ In this review, we have discussed strategies for targeting signaling pathways in PCa, as well as their mechanisms and related clinical trials.

## Genomic landscape and therapeutic targets

Inflammation and chronic prostatic diseases, which are possibly associated with diet, chemical injury, and microbial infection, are believed to drive prostate carcinogenesis through DNA damage and mutagenesis.^76,77^ The initiation and progression of PCa are linked to complex interactions between acquired somatic gene alterations and microenvironmental factors.^4,76,77^ Both hereditary and environmental factors can increase the risk of PCa. Estimations of the hereditary risk of PCa have been partly explained in the Nordic Twin Study of Cancer (which included 80,309 monozygotic and 123,382 same-sex dizygotic twins), which found that ~60% cases of PCa are influenced by genetic factors.^78^ Of environmental factors, smoking, alcohol consumption, and infections (such as gonorrhea and HPV) increase the risk of developing PCa.^79–82^ Furthermore, obesity and diet (such as the consumption of saturated animal fat and meat) are also associated with increased risk of PCa.^83–85^ Interestingly, incidence rates of PCa have large international geographical variations (for example, Australia/New Zealand has the highest incidence of PCa, almost 25 times higher than that in areas such as South-Central Asia), while immigrants moving from countries with lower PCa incidence to countries with higher PCa rates soon acquire higher risks,^86,87^ suggesting complex mechanisms for the etiologies of PCa.

**Fig. 1:**
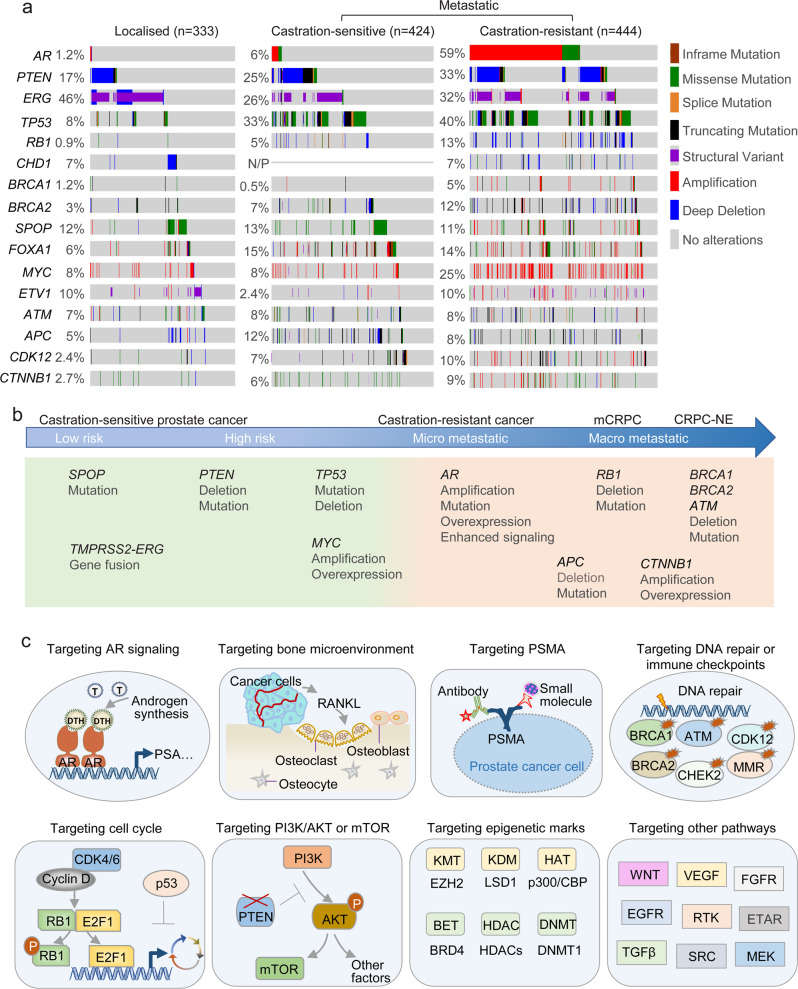
Overview of genetic alterations and therapeutic strategies in PCa. **a** Genetic alterations in localized PCa, metastatic castration-sensitive PCa, and metastatic castration-resistant PCa.^90–93^ **b** Common somatic mutations at different disease stages of PCa.^4^ **c** Overview of therapeutic targeting strategies for the treatment of PCa

Nevertheless, with the development of next-generation sequencing techniques, substantial advances have been made in understanding genomic alterations in PCa (Fig. 1a, b).^88–94^ In the early stage of PCa, frequent genomic alterations include *TMPRSS2-ERG* fusions in 40–60% of patients and *SPOP* mutations in 5–15% of patients.^88,90^ Interestingly, Asian patients with PCa have fewer *TMPRSS2-ERG* fusions, whereas genomic alterations in *FOXA1, ZNF292*, and *CHD1* are observed in more than 40% of these patients.^95^ Aberrations in *AR* are infrequent in the early stage of PCa, but AR pathway alterations and increased AR signaling commonly occur in advanced PCa via amplification, gain-of-function mutations, or overexpression or increased transcription of AR (Fig. 1a, b).^88,90,92^ Genomic alteration of *PTEN* and *TP53* often occurs across different stages of PCa (Fig. 1a, b).^92^ The proportion of *PTEN* and *TP53* deletions or mutations is 10–20% in localized PCa, but increases to nearly 40% in mCRPC (Fig. 1a, b).^88,90,92^ Oncogene *MYC* amplification or WNT signaling activation via *APC* loss and *CTNNB1* amplification are also frequent, occurring in approximately 10–30% of all mCRPC cases (Fig. 1a, b).^88,90,92^ *RB1* loss is seen in approximately 10% of cases in mCRPC, and has been associated with poor prognoses (Fig. 1a, b).^88,90,92^ The concurrence of *PTEN* deletion, *TP53* mutations, and *RB1* loss are correlated with lineage plasticity and neuroendocrine prostate cancer (NEPC), which is highly treatment-refractory.^88,89,96,97^ Aberrations in DNA damage response genes, such as *BRCA1*, *BRCA2*, *ATM*, *CHEK2*, and *CDK12*, occur in approximately 20% of metastatic PCa (Fig. 1a, b).^88,90,92^ The alterations in DNA damage response genes as well as mismatch repair (MMR) genes have led to efficiently targeted approaches, which will be discussed later. Given that genomic alterations and signaling activation are diverse in different stages of the disease as well as in individual patients, multiple approaches have been developed to target various pathways and practiced in clinical trials (Fig. 1c).

## Targeting AR signaling

### Androgen-signaling axis

**Fig. 2:**
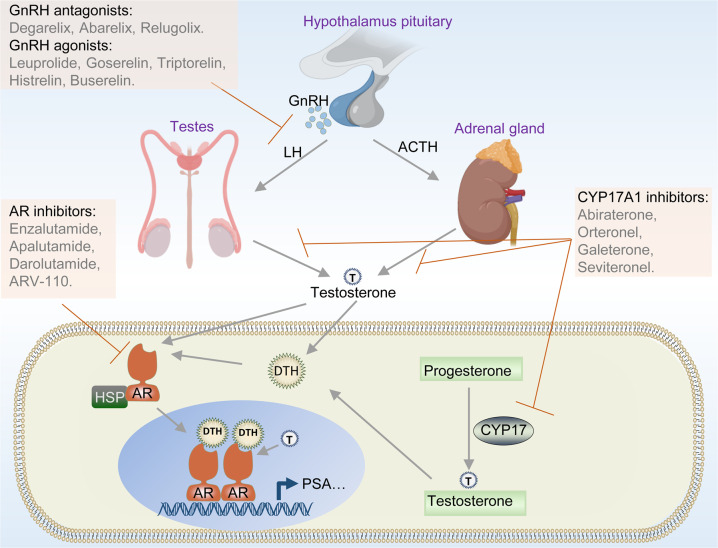
AR signaling pathway and targeted therapeutic approaches in PCa. Androgen synthesis is regulated by the hypothalamic–pituitary–gonadal axis. When androgens such as testosterone (T) and DHT bind to AR, AR releases itself from the heat shock protein complex, translocates into the nucleus, and promotes gene transcription to accelerate tumor progression. Targeting the androgen-signaling axis includes multiple approaches, such as targeting GnRH to prevent luteinizing hormone release, targeting CYP17A1 to restrain androgen synthesis, or directly targeting AR to inhibit AR transcription activity. Parts of images generated from BioRender (https://biorender.com/)

The androgen-signaling axis plays a crucial role in PCa progression.^7^ Androgen synthesis is tightly regulated by the hypothalamic–pituitary–gonadal axis (Fig. 2).^98,99^ When bound by androgens such as testosterone and dihydrotestosterone (DHT), AR releases from the heat shock protein complex, translocates into the nucleus, and promotes gene transcription to accelerate tumor progression, as well as maintains normal prostate cell maturation (Fig. 2).^100,101^ ADT has become essential in the treatment of PCa and metastatic disease since Huggins and Hodges first discovered the central role of the androgen-signaling axis in PCa after finding that orchiectomy significantly suppresses tumor progression.^102^ The aim of ADT by either orchiectomy or chemical castration is to suppress serum testosterone to castration levels and thus block the activation of the AR.^103^ So far, the most effective strategy to treat PCa is still to target the androgen-signaling axis, which includes multiple approaches, such as targeting GnRH to prevent luteinizing hormone release, targeting cytochrome P450 17α-hydroxylase/17,20-lyase (CYP17A1) to restrain androgen synthesis, or directly targeting AR to inhibit AR transcriptional activity (Fig. 2).^103–107^

### AR

AR, a steroid receptor transcriptional factor composed of 919 amino acids and encoded by the gene located on chromosome X (Xq11-12), is composed of an N-terminal domain (NTD) encoded by exon 1, a DNA-binding domain (DBD) encoded by exons 2–3, hinge region encoded by exon 4, and a ligand-binding domain (LBD) encoded by exons 5–6.^100,108^ AR plays an important role in PCa pathogenesis, and its expression has been found in most primary and metastatic PCa.^109,110^ The intensity of AR staining in the nucleus of bone mCRPC is associated with a worse outcome.^111,112^ The activation of AR signaling supports the survival and growth of PCa cells.^7,113^ Mechanically, in the absence of ligands such as DHT and testosterone, the AR is located in the cytoplasm and complexes with chaperone proteins such as HSP90. When the ligands bind to the LBD of AR, they translocate into the nucleus to form a homodimer, and the AR dimer interacts with its coregulatory proteins to recognize cognate DNA response elements located in the proximal or distal intragenic and intergenic regions of androgen target genes, thereby regulating gene expression (e.g., *KLK3*, *NKX3.1*, *FKBP5*, *TMPRSS2-ERG*).^105^

AR inhibitors (bicalutamide, flutamide, and nilutamide),^114–120^ which bind to the LBD of AR and result in the inhibition of androgen binding to LBD, reduce the serum level of PSA (encoded by the *KLK3* gene) and alleviate symptoms in PCa patients.^106^ Recently, several novel AR inhibitors have been developed and used in clinical settings (Table 2). Enzalutamide (also known as MDV3100),^121^ approved by the FDA in 2012, is a second-generation AR inhibitor with a high affinity for the LBD of AR. Multiple clinical trials have confirmed that enzalutamide significantly prolongs the overall survival of patients with metastatic or nonmetastatic CRPC.^13,14,122–124^ Apalutamide (also known as ARN-509)^125^ has a greater efficacy than enzalutamide and was approved for treatment of nonmetastatic CRPC by the FDA in 2018. Apalutamide inhibits the nuclear localization and DNA binding of AR in PCa cells.^125^ A clinical study showed that apalutamide administration significantly lengthened metastasis-free survival in patients with nonmetastatic CRPC.^15^

**Table 2:** Selective clinical trials of AR signaling inhibitors

Drug	Target	Condition	Status	Phase	NCT identifier
Degarelix	GnRHR	PCa	Completed	3	NCT00451958
Degarelix	GnRHR	PCa	Completed	3	NCT01071915
Degarelix	GnRHR	PCa	Completed	3	NCT02015871
Relugolix	GnRHR	PCa	Recruiting	3	NCT05050084
Relugolix	GnRHR	PCa	Completed	3	NCT03085095
Abarelix	GnRHR	PCa	Completed	3	NCT00841113
Leuprolide	GnRHR	PCa	Completed	4	NCT00220194
Leuprolide	GnRHR	PCa	Recruiting	3	NCT04914195
Goserelin	GnRHR	PCa	Completed	3	NCT00439751
Goserelin	GnRHR	PCa	Not yet recruiting	4	NCT03971110
Triptorelin	GnRHR	PCa	Completed	3	NCT01715129
Triptorelin	GnRHR	PCa	Completed	3	NCT00104741
Histrelin	GnRHR	Metastatic PCa	Recruiting	2/3	NCT04787744
Histrelin	GnRHR	PCa	Completed	3	NCT01697384
Buserelin	GnRHR	PCa	Completed	3	NCT00003653
Buserelin	GnRHR	PCa	Recruiting	3	NCT05050084
Enzalutamide	AR	CRPC	Completed	3	NCT00974311
Enzalutamide	AR	mCRPC	Completed	4	NCT02116582
Enzalutamide	AR	mCRPC	Completed	4	NCT02485691
Apalutamide	AR	PCa	Completed	2	NCT01790126
Apalutamide	AR	Nonmetastatic CRPC	Recruiting	3	NCT04108208
Darolutamide	AR	PCa	Not yet recruiting	3	NCT02799602
Darolutamide	AR	Nonmetastatic CRPC	Completed	3	NCT02200614
ARV-110	AR	mCRPC	Recruiting	1/2	NCT03888612
Abiraterone	CYP17A1	mCRPC	Completed	3	NCT04056754
Abiraterone	CYP17A1	mCRPC	Completed	3	NCT00887198
Abiraterone	CYP17A1	mCRPC	Completed	3	NCT02111577
Seviteronel	CYP17A1	CRPC	Completed	2	NCT02445976
Orteronel	CYP17A1	mCRPC	Completed	3	NCT01193257
Galeterone	CYP17A1	CRPC	Completed	2	NCT01709734

Notably, AR gene mutations and amplifications occur in ~60% of mCRPC (Fig. 1a). AR mutations are predominant in LBD, limiting binding affinity of AR inhibitors.^126–129^ Darolutamide (also named ODM-201),^130,131^ approved for treatment of nonmetastatic CRPC by the FDA in 2019, is a novel AR inhibitor that antagonizes mutated AR, such as F877L and T878A, which confers resistance to enzalutamide and apalutamide.^132–134^ Phase 3 trial studies have shown that darolutamide significantly prolongs metastasis-free survival for high-risk nonmetastatic CRPC.^16,135^ Furthermore, the latest AR protein degrader ARV-110, an oral proteolysis targeting chimera (PROTAC),^136^ specifically degrades more than 95% of AR and overcomes enzalutamide resistance in xenograft models.^137,138^ ARV-110 is presently under evaluation in clinical trials (Table 2). Current AR-targeted therapies primarily target LBD. However, AR variants such as AR-V7 and ARv567es lack the entire LBD or a functional LBD, but retain their ability to bind DNA in the absence of androgens and display constitutive activity, thus conferring drug resistance to next-generation AR inhibitors.^139–143^

### GnRH

GnRH, also known as luteinizing hormone-releasing hormone (LHRH),^144,145^ is a hypothalamic peptide (pGlu-His-Trp-Ser-Tyr-Gly-Leu-Arg-Pro-Gly-NH2) that plays a central role in controlling the hypothalamic–pituitary-axis in mammals.^146–148^ GnRH binds to the GnRH receptor (GnRHR), which belongs to the rhodopsin-like G protein coupled receptor (GPCR) family, and induces the release of luteinizing hormone (LH), which then arrives to the Leydig cells of the testes to stimulate testosterone synthesis.^148–151^ Schally and Guillemin (1982) first developed a synthetic GnRH agonist (also known as GnRH analog) to manipulate the hypothalamic–pituitary–gonadal axis.^152^ Mechanically, the GnRH agonists induce sustained stimulation of the pituitary gland to induce the downregulation and desensitization of GnRHR, resulting in the reduction of LH release and suppression of testosterone production to castration levels.^103,148,153^ An early study by Tolis et al. found that patients with advanced PCa treated daily with GnRH agonists experienced a 75% suppression in serum testosterone levels, resulting in a decreased prostate size and reduction in tumor-associated bone pain.^152^ Several synthetic GnRH agonists have been developed for clinical use since the 1980s, including leuprolide, triptorelin, and buserelin.^154–166^ Many clinical studies of GnRH agonists are completed or ongoing (Table 2). Although long-acting GnRH agonists suppress the release of LH and testosterone, GnRH agonists initially produce a rapid and transient increase in LH and testosterone levels, which is called the “flare-up” phenomenon, and may lead to side effects, such as bone pain and cardiovascular complications.^103,154^

In contrast to GnRH agonists, GnRH antagonists bind competitively to the GnRHR in the pituitary to rapidly prevent LH production, thereby suppressing testosterone to castration levels, which reduces the risk of the “flare-up” phenomenon.^103,167^ Abarelix was the first GnRH antagonist approved by the FDA, but it was discontinued from the market because of severe hypersensitivity reactions.^168–171^ Degarelix, which was approved by the FDA in 2008, is the most widespread GnRH antagonist used in clinical practice.^172–177^ Relugolix, which is a novel oral GnRH antagonist, was approved by the FDA in 2020.^178,179^ Numerous promising clinical trials on GnRH antagonists in combination with radiotherapy or chemotherapy have been completed or are still ongoing (Table 2). However, the use of these agents remains controversial. For example, a clinical study revealed that neoadjuvant degarelix is related to the upregulation of DHT in tumors.^180^ Other studies have found that the administration of GnRH agonists or antagonists can decrease lean body mass and increase fat mass.^181–183^ Further studies will provide critical evidence to address whether GnRH agonists or antagonists are safe for patients with cardiovascular disease.

### CYP17A1

CYP17A1, a membrane-bound monooxygenase, is a pivotal enzyme for androgen synthesis.^184^ CYP17A1 is composed of 508 amino acids with four structural domains, including a substrate-binding domain, a catalytic activity area, a heme-binding region, and a redox-partner binding site.^185,186^ CYP17A1 has both 17α-hydroxylase and 17,20-lyase catalytic activities, and is essential in the production of both androgens and glucocorticoids.^185,186^ CYP17A1 predominantly localizes at the endoplasmic reticulum in the adrenal glands, testicular Leydig cells, and ovarian thecal cells.^187^ The 17α-hydroxylase activity of CYP17A1 is required for the hydroxylation of pregnenolone and progesterone at the C17 position, which generates 17α-hydroxypregnenolone and 17α-hydroxyprogesterone.^188^ The 17,20-lyase activity of CYP17A1 is essential for the cleavage of 17α-hydroxypregnenolone or 17α-hydroxyprogesterone, which form dehydroepiandrosterone (DHEA) and androstenedione, respectively, and is a critical step for testosterone synthesis.^105,188^ Importantly, CYP17A1 also confers to intratumoral androgen biosynthesis in CRPC.^189–192^ Low levels of androgen are still found in the serum during ADT; thus, many CYP17A1 inhibitors have been tested in clinics (Table 2).

Abiraterone, a selective inhibitor of 17α-hydroxylase and 17,20-lyase,^193^ was first approved by the FDA for treatment of mCPRC in 2011.^194,195^ Administration of abiraterone induces a significant decline in PSA levels, improves overall survival, and alleviates pain in both chemotherapy-naive and docetaxel-resistant patients.^12,196–198^ Although the suppression of 17α-hydroxylase activity leads to overproduction of mineralocorticoids, which can result in adverse events (such as hypokalaemia, fluid retention, hypertension, and cardiac disorders), these side effects are largely prevented by the co-administration of glucocorticoid prednisone.^199^

Other CYP17A1 inhibitors, such as orteronel (TAK-700) and galeterone (TOK-001), have also been developed.^200–202^ Orteronel is a nonsteroidal selective inhibitor of 17,20-lyase, while galeterone has multiple mechanisms of action, including CYP17A1 inhibition, AR antagonism, and a reduction in both full-length AR and AR-V7 levels.^192,200–203^ Orteronel preferentially inhibits 17,20-lyase over 17α-hydroxylase, leading to a reduction in the risk of overproduction of mineralocorticoids.^184,192,201^ Results from phase 1/2 studies have indicated that patients had an approximately 60% PSA response rate at 12 weeks after the administration of orteronel twice daily.^204^ A phase 1 study in patients with CRPC observed that ~50% of men had a PSA decline after 12 weeks of treatment with galeterone, and no adrenal mineralocorticoid excess was noted.^205^ Therefore, orteronel and galeterone are potentially attractive drugs for longer duration therapy and overcoming drug resistance, although a clinical study showed that orteronel did not meet the primary endpoint of overall survival.^206^ Clinical studies of galeterone compared to enzalutamide in mCRPC expressing AR-V7 have been conducted, but the result do not meet the primary endpoint.^207^ Additionally, seviteronel (VT-464), a newly developed drug used as a CYP17A1 inhibitor and AR antagonist, selectively inhibits 17,20-lyase and greatly decreases AR transactivation and offers an advantage over abiraterone because it does not require combination with prednisone.^208^ Notably, abiraterone treatment markedly increases intratumoral expression of CYP17A1 in tumor biopsies from CRPC patients, and many patients ultimately become resistant to CYP17 inhibitors.^143,190,209^

## Targeting bone microenvironment

### Bone microenvironment of PCa

**Fig. 3:**
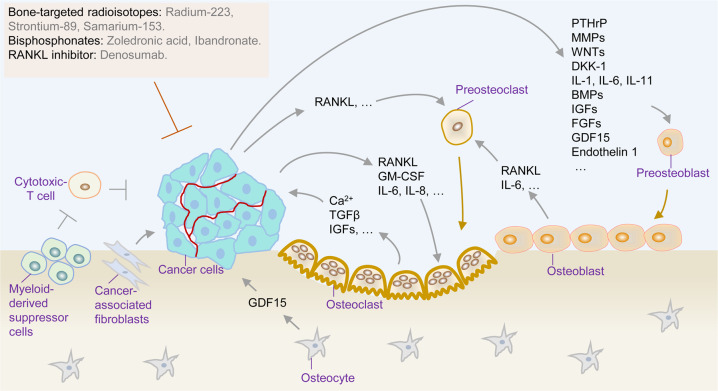
The vicious cycle of bone metastasis and targeting strategies in PCa. Tumor cells in bone secrete osteolytic cytokines such as RANKL, PTHrP, GM-CSF, MMPs, IL-6, IGFs, FGFs, endothelin 1, GDF15, DKK-1, and WNTs to induce osteoclast-mediated bone resorption, while osteoclasts release bone-stored factors such as TGFβ, IGFs, and Ca^2+^ that stimulate tumor cell proliferation, establishing a vicious cycle. Targeting the bone microenvironment (such as bone-targeted radioisotopes, bisphosphonates, and RANKL inhibitors) is effective to manage bone metastases in PCa

Bone metastasis in PCa is a highly frequent event that occurs in up to 90% of patients with advanced disease.^210–212^ The bone microenvironment is a dynamic compartment that provides a milieu in which metastatic cancer cells can colonize and grow.^213,214^ The “vicious cycle” hypothesis is an appropriate model with which to explain the process of cancer cells metastasizing to the bone (Fig. 3).^213,215^ Tumor cells in bone induce osteoclast-mediated bone resorption, while osteoclasts release bone-stored factors that stimulate tumor cell proliferation, establishing a vicious cycle. Bone metastasis is driven by the cooperation among metastatic tumor cells, bone-forming osteoblasts, bone-dissolving osteoclasts, and other cell populations.^212^ Physiologically, mature osteoblasts, osteocytes, and osteoclasts regulate the dynamic remodeling of bone tissue.^216^ The increased levels of parathyroid hormone-related peptide (PTHrP) from osteoclasts can induce bone resorption by upregulating the receptor activator of RANKL, which promotes the release of various growth factors (such as ionized calcium and TGFβ) into the bone microenvironment to support cancer cell implantation and transformation.^217–221^ Invading tumor cells secrete osteolytic cytokines, such as granulocyte-macrophage colony-stimulating factor, matrix metalloproteinases, interleukin (IL)-6, insulin-like growth factors (IGFs), fibroblast growth factors (FGFs), endothelin 1, growth differentiation factor 15 (GDF15), dickkopf-1 (DKK-1), and WNTs.^222–226^ These osteolytic cytokines stimulate preosteoblast differentiation and promote osteoclast maturation to accelerate bone resorption.^217,227,228^ Meanwhile, osteoblasts release IL-6 and RANKL to accelerate the maturation of osteoclasts, further secreting growth factors to facilitate tumor cell growth (Fig. 3).^212,217,229^ Thus, various approaches that target the bone microenvironment, such as bone-targeting agents, are effective for managing bone metastases in PCa (Fig. 3).

### Bone-targeted radioisotopes

Bone-seeking therapeutic radioisotopes are distinct among anticancer therapies, because they target calcium hydroxyapatite in the bone instead of tumor cells. Bone metastases often contain osteosclerotic lesions, with increased osteoblastic bone formation. Thus, ionizing radiation is selectively delivered to bone with increased osteoblastic activity to simultaneously target multiple metastases,^230^ enabling the delivery of high-energy radiation to bone metastases while limiting toxicity to other normal cells. Calcium-mimetic radiopharmaceuticals, such as the first generation of primarily β-emitting radioisotopes strontium-89 and samarium-153, have been approved early by the FDA based on successful endpoints of bone pain palliation.^231–235^ More recently, the bone-targeted radioisotope radium-223 was also approved by the FDA for patients with mCRPC and painful bone metastases.^17,18,236–238^ Unlike strontium-89 and samarium-153, radium-223 is predominately an α-emitter with short tissue penetration range,^230^ which potentially reduces bone marrow toxicity and limits undue exposure.^237^ Radium-223 targets bone as a calcium-mimetic and preferentially uptakes into areas of increased bone formation, resulting in a highly localized antitumor effect on adjacent bone metastases.^239^ In a phase 3 clinical trial involving 921 CRPC patients with bone pain symptoms, administration of radium-223 reduced bone pain and significantly prolonged overall survival compared to patients administered placebo.^17^ Treatment with radium-223 was tolerated, although the frequency of thrombocytopenia was increased.^240^ Moreover, no patients with leukemia or other cancers were identified during long-term surveillance.^241^ Many promising results have been found in clinical settings, and further clinical trials of bone-targeted radioisotopes are still ongoing (Table 3).

**Table 3:** Selective clinical trials of drugs targeting bone microenvironment

Drug	Target	Condition	Status	Phase	NCT identifier
Radium-223	Calcium-mimetic α-emitting	Bone-metastatic CRPC	Completed	3	NCT00699751
Radium-223	Calcium mimetic α-emitting	Bone-metastatic CRPC	Completed	3	NCT01618370
Radium-223	Calcium mimetic α-emitting	Bone-metastatic CRPC	Completed	3	NCT01810770
Radium-223	Calcium mimetic α-emitting	Bone-metastatic CRPC	Recruiting	3	NCT03432949
Strontium-89	Calcium mimetic β-emitting	Bone-metastatic CRPC	Completed	3	NCT00002503
Samarium-153	Calcium mimetic β-emitting	Bone-metastatic CRPC	Completed	3	NCT00365105
Zoledronic acid	FDPS	Bone-metastatic CRPC	Completed	3	NCT00242567
Zoledronic acid	FDPS	Bone-metastatic CRPC	Completed	3	NCT00079001
Zoledronic acid	FDPS	Bone-metastatic CRPC	Completed	3	NCT00321620
Zoledronic acid	FDPS	Bone-metastatic CRPC	Completed	4	NCT00242554
Clodronic acid	FDPS	PCa	Completed	Unknown	NCT01198457
Risedronic acid	FDPS	PCa	Completed	3	NCT00426777
Alendronic acid	FDPS	Metastatic PCa	Terminated	2	NCT00019695
Ibandronic acid	FDPS	Metastatic PCa	Completed	3	NCT00082927
Denosumab	RANKL	CRPC	Completed	3	NCT01824342
Denosumab	RANKL	CRPC	Completed	3	NCT00286091
Denosumab	RANKL	CRPC	Completed	3	NCT00838201
Denosumab	RANKL	PCa	Completed	3	NCT00925600
Mipsagargin	SERCA	Metastatic PCa	Withdrawn	2	NCT01734681
SOR-C13	TRPV6	Advanced cancers	Recruiting	1	NCT03784677

### Bisphosphonates

The antitumor effects of bisphosphonates might be attributable to their anti-osteoclast activity.^212^ Bisphosphonates preferentially bind to hydroxyapatite of bone, resulting in increased uptake of bisphosphonates by osteoclasts during the osteoclastic resorption process.^242–244^ Because bisphosphonates are accumulated in bone, they are internalized selectively by osteoclasts rather than other cell types.^245^ Following uptake by osteoclasts, bisphosphonates intracellularly inhibit farnesyl diphosphate synthase (FDPS), which is a key enzyme for cholesterol biosynthesis. As a result, osteoclasts accumulate intracellular isopentenyl pyrophosphates, which form cytotoxic ATP analogs that induce the apoptosis of osteoclasts.^246^ In contrast, by the inhibitory effect on FDPS, bisphosphonates also inhibit the function of Rho GTPases by disrupting prenylation-dependent signaling,^244^ thus leading to the apoptosis of osteoclasts due to the impaired mobility and adhesion of these cells.^247^ Therefore, the exposure of osteoclasts to bisphosphonates leads to less bone resorption and lower release of bone-stored factors, breaking the “vicious cycle” between tumors and bone.^247^ The clinical value of bisphosphonate-based drugs (such as ibandronate,^248,249^ clodronate,^250,251^ pamidronate,^252,253^ and zoledronate^19,22,254–256^) has been shown in numerous trials of PCa (Table 3).

The third-generation bisphosphonate zoledronic acid has the highest affinity for bone and it was approved by the FDA to prevent skeletal-related events (SREs) in patients with mCRPC in 2002.^257,258^ A phase 3 study demonstrated that the zoledronic acid-treated group had fewer SREs compared to those in the placebo group (44.2% versus 33.2%, *p* = 0.021).^258^ Zoledronic acid also reduced the ongoing risk of SREs by 36% (risk ratio = 0.64, *p* = 0.002).^259^ However, bisphosphonates are also associated with adverse events. For example, a few patients receiving bisphosphonates developed hypocalcemia, nausea, emesis, diarrhea, gastric pain, esophagitis, gastrointestinal bleeding, or ulcers.^260,261^ In particular, intravenous administration of bisphosphonates is associated with an increased risk of renal impairment.^261–265^

### RANKL

RANKL, together with its receptor RANK and the decoy receptor osteoprotegerin, are key factors that regulate osteoclast development and bone metabolism.^266–269^ RANKL/RANK signaling induces preosteoclast differentiation and maintains the survival and function of osteoclasts.^270–273^ RANKL plays important role in bone metastases; therefore, a specific RANKL antibody denosumab that neutralizes the activity of RANKL has been developed.^274^ Denosumab has shown significant efficacy in inducing osteoclast apoptosis and impairing osteoclast activity.^275^ Accordingly, denosumab has been approved by the FDA for the treatment of diseases driven by a high activity of osteoclasts, including cancer with bone metastases as well as osteoporosis.^276–279^ Many clinical trials of denosumab have been conducted in PCa (Table 3). Denosumab significantly reduced SREs such as pathologic fractures and hypercalcemia. A phase 3 study enrolled 1432 men with nonmetastatic CRPC and a high risk of bone metastasis, and demonstrated that treatment of denosumab significantly improved bone-metastasis-free survival compared with placebo group (median 29.5 versus 25.2 months; *p* = 0.028), although it did not improve overall survival.^280^ Another trial involving 1904 patients found that denosumab treatment increased the median time to first on-study SREs compared with the results of zoledronic acid (20.7 versus 17.1 months; *p* = 0.008).^22^ Because denosumab treatment is associated with life-threatening hypocalcemia, proactive treatment of calcium and calcitriol should be considered when using denosumab.^281^

### Calcium channels

Cytosolic calcium (Ca^2+^) signaling plays an important role in the bone metastasis of PCa.^219^ Elevated Ca^2+^ stimulates PTHrP secretion and activates RANKL/RANK signaling in osteoclasts, which promotes bone resorption and calcium release, in turn promoting tumor cell proliferation and maintaining PCa cell homing to bone.^219^ Targeting calcium signaling could be a promising strategy for managing PCa bone metastasis. New agents for targeting calcium signaling include calcium-ATPase inhibitors, voltage-gated calcium channel inhibitors, transient receptor potential (TRP) channel inhibitors, and Orai inhibitors, although most of these agents are still in the early stages of studies.^282^ For instance, mipsagargin (G-202), a SERCA inhibitor, was tested in mCRPC in a phase 2 clinical trial; however, the study was withdrawn without results posted (Table 3). The TRPV6 inhibitor, SOR-C13, is currently under evaluation in patients with advanced tumors, including PCa, in a phase 1 clinical trial (Table 3). Because calcium channels are also critical for numerous cellular homeostasis and physiological functions under normal conditions,^283^ future calcium-based therapies should specifically target PCa cells to decrease normal tissue toxicity.

## Targeting PSMA

### PSMA and PSMA-targeted ligands

PSMA is a type II transmembrane glycoprotein that includes activities of folate hydrolase and N-acetyl-α-linked acidic dipeptidase and consists of 750 amino acids located in three domains, including the intracellular domain, which contains 19 amino acids, the transmembrane domain, which consists of 24 amino acids, and the extracellular domain, which contains 707 amino acids.^284,285^ PSMA is expressed at a very low level in normal prostatic tissues and nonprostatic tissues, but its expression in PCa tissues increases by 100–1000 times compared to that in normal tissues.^286^ Thus, PSMA is a theranostic target for imaging diagnostics and targeted radionuclide therapy for PCa and its metastases.^287–289^

Three main types of ligands are used to target PSMA: monoclonal antibodies, aptamers, and small-molecule inhibitors. PSMA monoclonal antibodies can be classified into two types: intracellular domain antibodies (7E11, PM2J004.5) and extracellular domain antibodies (J591, J533, J415).^284,290,291^ Importantly, the humanized monoclonal antibody J591, which targets the extracellular domain of PSMA, has an impressive application prospect in the diagnosis and treatment of PCa. Aptamers of PSMA (such as xPSM-A9, xPSM-A10, A10-3.2, and A9g) are nucleotides or deoxynucleotides that can selectively recognize PSMA.^292–294^ Small-molecule inhibitors that can interact with PSMA, including 123I-MIP-1072, 123I-MIP-1095, PSMA-I&T, PSMA-I&S, and PSMA-617, have become the preferred choice for molecular imaging probes and targeted therapy for PCa.^295,296^

### PSMA-based diagnostic imaging

The early PSMA-targeted imaging agent was ProstaScint (also known as ^111^In-capromab pendetide), a mouse monoclonal antibody (7E11) linked to ^111^In for SPECT (single photon emission computed tomography) imaging. However, ProstaScint was only able to bind to the intracellular epitope of PSMA, and cannot be accessed in viable tumor cells, thus limiting its clinical performance.^297,298^ ^68^Ga-PSMA-11 (also called PSMA-HBED-CC) for PET is probably the tracer most often used for PCa.^299–301^ Several ^68^Ga-labeled PSMA ligands have been developed as theranostic agents, including ^68^Ga-PSMA-617 and ^68^Ga-PSMA-I&T.^302–304^ The ^18^F-labeled agents include ^18^F-DCFBC, ^18^F-DCFPyL, and ^18^F-PSMA-1007, which exhibit many advantages such as a lower positron range and longer half-life compared with those of ^68^Ga-labeled agents.^305–308^ PSMA-based imaging has shown improved sensitivity and diagnostic accuracy in PCa. For example, a study in 96 patients with PCa demonstrated that ^18^F-PSMA-1007 PET had a sensitivity of 85.9% and a specificity of 99.5% in a patient-based analysis for detecting positive lymph nodes larger than 3 mm.^309^ Furthermore, the ^99m^Tc-labeled PSMA ligand ^99m^Tc-MIP-1404 is in a phase 3 clinical trial designed to evaluate its sensitivity and specificity to detect PCa (Table 4). Standardized criteria of the PSMA ligand PET are evolving and will facilitate its use in clinical practice; several prospective trials (Table 4) are ongoing to support final market approval.^310^

**Table 4:** Selective clinical trials of PSMA-targeted agents

Drug	Target	Condition	Status	Phase	NCT identifier
^177^Lu-PSMA-617	PSMA	mCRPC	Unknown	2	NCT03392428
^177^Lu-PSMA-617	PSMA	mCRPC	Recruiting	3	NCT04689828
^225^Ac-PSMA-617	PSMA	PCa, CRPC	Recruiting	1	NCT04597411
^177^Lu-J591	PSMA	Metastatic PCa	Completed	2	NCT00195039
MLN2704	PSMA	mCRPC	Completed	1/2	NCT00070837
PSMA-MMAE	PSMA	mCRPC	Completed	2	NCT01695044
MEDI3726	PSMA	mCRPC	Completed	1	NCT02991911
BIND-014	PSMA	mCRPC	Completed	2	NCT01812746.
^68^Ga-PSMA-11	PSMA	PCa	Completed	3	NCT03803475
^68^Ga-PSMA-617	PSMA	PCa	Completed	2	NCT03604757
^18^F-PSMA-1007	PSMA	PCa	Completed	3	NCT04102553
^99m^Tc-MIP-1404	PSMA	PCa	Completed	3	NCT02615067
CART-PSMA-TGFβRDN	PSMA	mCRPC	Not yet recruiting	1	NCT04227275
PD1-PSMA-CART	PSMA	CRPC	Recruiting	1	NCT04768608
LIGHT-PSMA-CART	PSMA	CRPC	Suspended	1	NCT04053062
P-PSMA-101 CAR-T	PSMA	PCa	Recruiting	1	NCT04249947

### PSMA-targeted radionuclide therapy (PSMA-TRT)

In contrast to conventional external radiotherapy, targeted radionuclide therapy (TRT) is a treatment conducted by injecting a radionuclide-labeled ligand into the body to specifically target cancer cells. The radionuclide then releases α-particles, β-particles, or auger electrons to produce free radicals that induce DNA damage, thus specifically promoting apoptosis or necrosis of targeted cells.^284,311^ Conjugations of PSMA-targeted ligands (antibodies or small molecules) with radionuclides such as β-emitters (most commonly ^177^Lu) or α-emitters (commonly ^225^Ac) produce PSMA-TRT agents, including ^177^Lu-PSMA-617, ^225^Ac-PSMA-617, and ^177^Lu-J591 (Table 4).^312–314^ More importantly, in a recent phase 2 study comparing ^177^Lu-PSMA-617 and cabazitaxel in mCRPC, ^177^Lu-PSMA-617 led to a higher PSA response (66% versus 44% by intention-to-treat analysis; *p* = 0.0016) and fewer adverse events, indicating that ^177^Lu-PSMA-617 is a potential alternative therapy to cabazitaxel in patients with mCRPC.^315^ Lu-PSMA-617 was approved by the FDA for the treatment of mCRPC in 2021. In addition to ^177^Lu-PSMA-617, ^225^Ac-PSMA-617 and ^177^Lu-J591 have been studied in clinical trials (Table 4). Ongoing clinical trials of PSMA-TRT will further explore the optimal sequencing of this therapy in earlier disease settings, as well as novel combinations. Overall, PSMA expression at different metastatic sites among different patients and the selection of optimal patients remain to be defined.

### PSMA-antibody-drug conjugates (PSMA-ADC)

PSMA-specific antibodies have been used to bind cytotoxic drugs via different chemical bonds to obtain PSMA-ADC. PSMA-ADC avoids systemic medication and reduces the toxicity to non-target organs compared to traditional cytotoxic drugs. Many applications of monoclonal antibody-based PSMA-ADC have entered clinical trials (Table 4), including that of MLN2704, which links to the antimicrotubule agent maytansinoid-1;^316^ PSMA-MMAE (monomethyl auristatin E), which connects to the microtubule disrupting agent MMAE;^317^ MEDI3726, which combines with the pyrrolobenzodiazepine-based linker-drug tesirine;^318^ and BIND-014, which conjugates with docetaxel.^40^ MLN2704 was discontinued after a phase 1/2 study because of the instability of the bond between the antibody and the drug.^319,320^ The first clinical trial of MEDI3726 observed a high incidence of treatment-related adverse events.^321^ A phase 2 clinical trial demonstrated that PSMA-MMAE showed some activity with regards to PSA decline and circulating tumor cell reduction in patients with mCRPC, but it also included significant treatment-related toxicities, such as neutropenia and neuropathy.^317^ Interestingly, the phase 2 clinical trial of BIND-014 (a novel PSMA-ADC) in patients with chemotherapy-naive mCRPC suggest that BIND-014 is well tolerated and patients are likely to benefit from the treatment.^40^ Optimization of dose administration, conjugation of more appropriate drugs, and patient selection should be considered to improve the efficacy of PSMA-ADC in the future.

### PSMA-based chimeric antigen receptor (CAR)-T cells therapy

CAR-T cells are genetically engineered T cells that express an artificial T-cell receptor, endowing T-cell populations with the ability to target tumors independently of major histocompatibility-complex (MHC) engagement.^322^ CAR-T-cell therapy has gained momentum in PCa treatment in clinical trials. CAR-T cells are activated when the antigen is recognized by CAR, thus stimulating the release of cytotoxins, such as perforin and granzyme, into tumor cells to induce apoptosis. PSMA is considered to be a reliable target for CAR-T-cell therapy. First-generation CAR-T cells targeting PSMA were constructed with a chimeric anti-PSMA immunoglobulin-T-cell receptor gene based on the monoclonal antibody 3D8.^323^ Second-generation CAR-T cells were constructed by inserting the CD28 signal domain into first-generation CAR-T cells.^324^ Recently, many new PSMA-based CAR-T cells, such as CART-PSMA-TGFβRDN, have been evaluated in phase 1 clinical trials in CRPC patients.^42^ Meanwhile, other clinical trials are underway and will test the safety and efficacy of PSMA-targeted CAR-T cells for the treatment of PCa (Table 4). Additionally, side effects such as cytokine release syndrome, immune effector cell-associated neurotoxicity syndrome, and cytopenia are commonly observed in patients receiving CAR-T-cell therapy; therefore, additional treatment with corticosteroids should be considered when using CAR-T-cell treatment.^325^

## Targeting DNA repair pathways

### PARP function in DNA repair and synthetic lethality

**Fig. 4:**
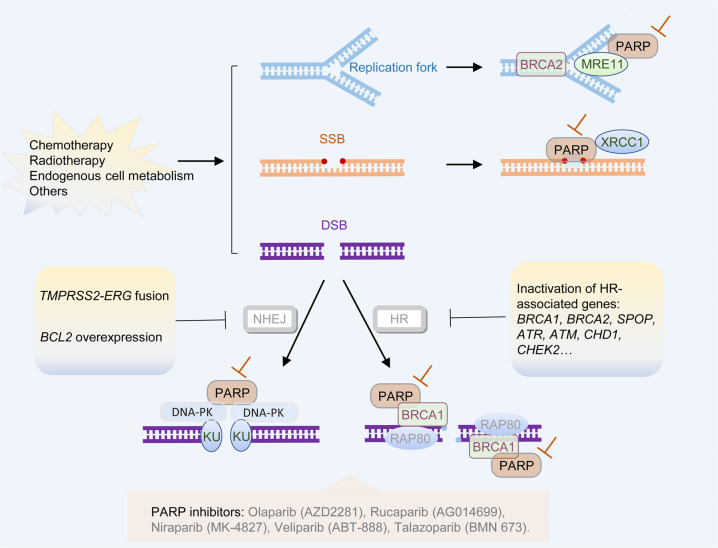
Inhibition of PARP mediates synthetic lethality in PCa. When PARP1/2 are pharmacologically inhibited, the accumulation of SSBs by PARP inhibition can progress to DSBs, which are usually repaired through HR. The DSBs can be fixed if the DNA repair system is intact in cells; however, PARP inhibition can lead to lethality if a cell is lacking HR repair capacity (mutations of BRCA1, BRCA2, or ATM). *BCL2* overexpression, *TMPRSS2-ERG* fusion, *SPOP* mutation, *PTEN* loss, and *CHD1* deletion are also linked with an impaired DNA damage response phenotype, which might increase the therapeutic effectiveness of PARP inhibition

PARP is a family of enzymes involved in DNA repair and transcriptional regulation.^326^ Activation of PARP1/2 is important for recruiting the key effectors of DNA repair (Fig. 4).^327^ DNA damage in cells, including single-strand breaks (SSB) and double-strand breaks (DSB), can be induced by exposure to chemicals (such as chemotherapy), physical agents (such as radiotherapy), or endogenous reactive metabolites (such as reactive oxygen and nitrogen species) (Fig. 4). Effective DNA repair is essential for cellular survival. Mechanisms of SSB repair include base-excision repair, nucleotide excision repair, and mismatch excision repair, whereas DSB repair includes homologous recombination (HR) and non-homologous end-joining (NHEJ).^327^ The primary mechanism for inhibiting PARP in cancer therapy is synthetic lethality, which indicates two genomic alteration events that are each relatively innocuous individually but become lethal when they occur together.^328^ When PARP1/2 is pharmacologically inhibited, the accumulation of SSB by PARP inhibition can progress to DSB, which is usually repaired through HR. The DSBs can be fixed if the DNA repair system is intact in cells; however, PARP inhibition would lead to lethality if a cell lacks HR repair capacity (mutations of *BRCA1*, *BRCA2*, or *ATM*).^329^ PARP inhibition would not induce cell death in normal cells due to efficient DSB repair mechanisms; however, PARP inhibition would be lethal for tumor cells with deficient HR, such as *BRCA1/2* mutations.^330–332^ Furthermore, PARP inhibition would result in fork collapse and would transform into DSB, since PARP1 is involved in the restart of stalled forks.^333,334^ If the function of the BRCA (breast cancer susceptibility protein) is deficient, these DSB would not be repaired, thus causing synthetic lethality. Up to 30% of mCRPC tumors harbor DNA damage repair gene aberrations,^24^ which can be therapeutically used with PARP inhibitors to induce synthetic lethality. However, the interpretation of PARP inhibition-related mechanisms of synthetic lethality may be incomplete. PARP inhibitors may also induce cytotoxic effects by inhibition of SSB repair, as well as other mechanisms.^335^ Moreover, genomic alterations, such as *TMPRSS2-ERG* fusion, *SPOP* mutation, *PTEN* loss, and *CHD1* deletion, are linked to an impaired DNA damage response phenotype, which might increase the therapeutic effectiveness of PARP inhibition.^336^ DNA damage response genes are regulated by AR; consequently, the ADT response is also influenced by DNA repair deficiency.^337^ Functional inactivation of DNA repair pathways also enhances sensitivity to chemotherapy and radiotherapy, and this effect is further enhanced by inhibitors of the targeting DNA repair pathways that induce synthetic sensitivity or lethality in DNA repair-deficient cancers (Fig. 4).^336^

### PARP inhibitors

Several PARP inhibitors have been evaluated in clinical trials (Table 5). In 2020, olaparib was approved by the FDA for the treatment of mCRPC with deficient HR genes. The first clinical data from a phase 2 study demonstrated that 88% of patients with a homozygous deletion or mutation in gene-associated DNA repair responded to olaparib.^23^ Responses were observed in patients with deletions or mutations of *BRCA1*, *BRCA2*, *FANCA*, *CHEK2*, and *PALB2*. The overall survival in patients with *BRCA1/2* alterations are more favorable than in those who were negative (13.8 versus 7.5 months; *p* = 0.05).^23^ Rucaparib was approved by the FDA in 2020 based on a recent clinical study, which involved 78 mCRPC patients with DNA repair gene alterations, including *ATM* (*n* = 49), *CDK12* (*n* = 15), *CHEK2* (*n* = 12), and other genes (*n* = 14).^338^ A PSA response was seen in 54.8% of all patients, and those that had *PALB2*, *FANCA*, *BRIP1*, and *RAD51B* mutations showed a better response compared with those with *ATM* alterations, while the objective response rate and PSA responses in patients with *ATM*, *CHEK2*, and *CDK12* mutations were low compared to those with *BRCA* mutated tumors.^338^ The efficacy of rucaparib is currently being evaluated in a phase 3 trial (Table 5). A phase 2 study demonstrated that the objective response rate of talazoparib was seen in 29.8% (31 of 104) of patients, whereas serious treatment-related adverse events were reported in 43 (34%) patients.^27^ In addition, PARP1/2 selective inhibitors, including niraparib and pamiparib, are currently being tested in phase 2/3 trials of PCa (Table 5). Finally, trials combining PARP inhibitors with other drugs, such as AR-targeting agents and radium-223, have gained momentum based on the concept of cross-sensitivity. For example, a randomized trial combining veliparib and abiraterone determined that the subgroup patients (27%) with aberrations in DNA repair genes showed better response rates to the combination group compared to those with abiraterone alone.^339^ However, the identification of prognostic and predictive biomarkers of responses in combination trials remains difficult.

**Table 5:** Selective clinical trials of PARP inhibitors

Drug	Target	Condition	Status	Phase	NCT identifier
Olaparib (AZD2281)	PARPs	PCa	Completed	1	NCT02324998
Olaparib (AZD2281)	PARPs	mCRPC	Not yet recruiting	3	NCT03732820
Olaparib (AZD2281)	PARPs	mCRPC	Not yet recruiting	3	NCT02987543
Olaparib (AZD2281)	PARPs	mCRPC	Not yet recruiting	3	NCT03834519
Rucaparib (AG014699)	PARPs	HR deficient mCRPC	Completed	2	NCT02952534
Rucaparib (AG014699)	PARPs	mCRPC	Not yet recruiting	3	NCT02975934
Veliparib (ABT-888)	PARPs	mCRPC	Completed	2	NCT01576172
Talazoparib (BMN 673)	PARPs	HR deficient mCRPC	Not yet recruiting	2	NCT03148795
Talazoparib (BMN 673)	PARPs	mCRPC	Recruiting	3	NCT03395197
Niraparib (MK-4827)	PARP1/2	mCRPC	Completed	1	NCT02924766
Niraparib (MK-4827)	PARP1/2	mCRPC	Recruiting	3	NCT04497844
Pamiparib (BGB-290)	PARP1/2	mCRPC	Terminated	2	NCT03712930
Pamiparib (BGB-290)	PARP1/2	mCRPC	Not yet recruiting	1	NCT03150810

## Targeting immune checkpoints

### MMR defect and immunotherapy response

**Fig. 5:**
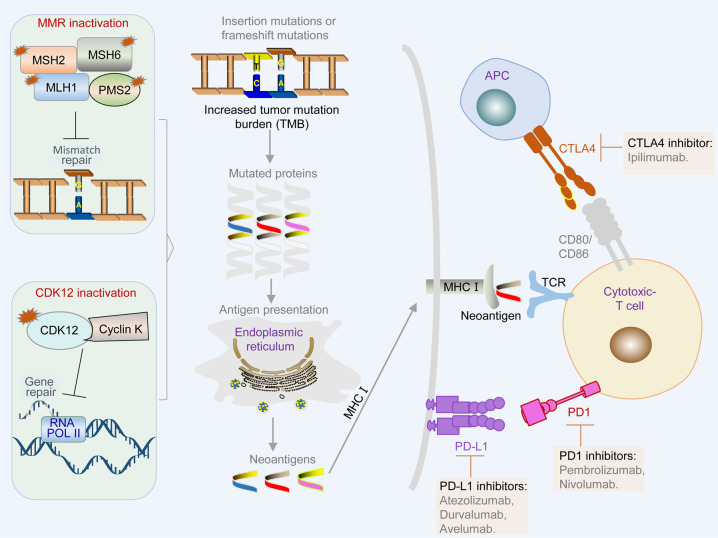
Mechanisms of elicitation of T-cell-mediated cancer killing in MMR- or CDK12-deficient cancer cells. A dysfunctional MMR system or CDK12 generate neoantigens through mutant peptides, or frameshift mutations and indels in coding microsatellites. These neoantigens are presented to the cell surface by MHCI molecules, thus facilitating T-cell-mediated tumor cell killing, which can be enhanced by ICIs, such as CTLA4 inhibitors, PD1 inhibitors, and PD-L1 inhibitors

Tumors with defects in MMR genes or microsatellite instability (MSI) often have an enhanced antitumor immune response, displaying a higher density of tumor-infiltrating lymphocytes (TILs).^340,341^ This phenomenon is attributed to high rates of mutations and increased levels of neoantigens in MMR-deficient tumors, which occur through different mechanisms, including mutant peptides, frameshift mutations, and indels in coding microsatellites.^342,343^ These neoantigens are presented on the cell surface by MHCI molecules, facilitating T-cell-mediated tumor cell killing (Fig. 5). In mammalian cells, MutL homolog 1 (MLH1), MutS homologue 2 (MSH2), MutS homologue 6 (MSH6) and PMS1 homologue 2 (PMS2) are the main proteins of the DNA MMR system, which are critical for recognizing and repairing erroneous insertion, deletion, and misincorporation of bases during DNA replication or DNA recombination.^344^ Approximately 3–5% of PCa cases are associated with deficiency of MMR genes, such as *MSH2*, *MSH6*, *PMS2*, and *MLH1*, resulting in hypermutation and MSI.^345^ Mutation of MMR genes in PCa is highly associated with increased expression of neoantigens and accumulation of TILs.^346^

### Immune checkpoint inhibitors (ICIs)

Several clinical studies have evaluated the efficacy of ICIs, including PD-L1 (nivolumab, atezolizumab, durvalumab, and avelumab), PD1 (pembrolizumab and nivolumab), and CTLA4 (ipilimumab) antibodies (Fig. 5 and Table 6). Early studies showed that ICIs exhibit limited anticancer activity.^347,348^ It is currently accepted that the selection of patients with deficiency in MMR genes is important, because this subset of patients is potentially responsive to ICIs.^345^ The anti-PD1 antibody pembrolizumab is approved by the FDA to treat cancers, including PCa with MMR mutations or MSI.^349^ The responses of ICIs in MMR mutations or MSI PCa are not universal. For example, ~54% (6 of 11) CRPC patients with MMR mutations or MSI-high tumors achieved a 50% reduction in PSA level after treatment with pembrolizumab.^345^ It remains unclear why some patients with MMR loss/MSI-high do not respond to ICI therapy in PCa. Despite these disappointing results, the interest in combining ICIs with other therapies remains high. Promising results were observed from a phase 1/2 clinical trial of pembrolizumab plus docetaxel, AR inhibitors, or PARP inhibitors in patients with mCRPC.^350^ Moreover, several phase 3 clinical trials to evaluate the efficacy of pembrolizumab combined with docetaxel, enzalutamide, and olaparib are ongoing (Table 6).

**Table 6:** Selective clinical trials of immune checkpoint inhibitors

Drug	Target	Condition	Status	Phase	NCT identifier
Ipilimumab	CTLA4	mCRPC	Completed	2	NCT02279862
Ipilimumab	CTLA4	mCRPC	Completed	3	NCT01057810
Ipilimumab	CTLA4	mCRPC	Completed	3	NCT00861614
Atezolizumab	PD-L1	mCRPC	Completed	1	NCT03024216
Atezolizumab	PD-L1	mCRPC	Recruiting	3	NCT04446117
Durvalumab	PD-L1	mCRPC	Completed	2	NCT03204812
Avelumab	PD-L1	NEPC	Completed	2	NCT03179410
Pembrolizumab	PD1	mCRPC	Recruiting	1/2	NCT02861573
Pembrolizumab	PD1	mCRPC	Completed	2	NCT03473925
Pembrolizumab	PD1	Hormone-sensitive PCa	Recruiting	3	NCT04934722
Pembrolizumab	PD1	mCRPC	Recruiting	3	NCT03834493
Pembrolizumab	PD1	mCRPC	Recruiting	3	NCT04907227
Nivolumab	PD1	mCRPC	Completed	2	NCT02601014
Nivolumab	PD1	mCRPC	Recruiting	3	NCT04100018
Ipilimumab + Nivolumab	CTLA4, PD1	mCRPC with CDK12 mutations	Recruiting	2	NCT03570619

Notably, up to 10% of mCRPC patients present with CDK12 aberration (Fig. 1a), which is associated with the response to ICIs.^351^ CDK12, which forms a complex with cyclin K, is critical for DNA repair during gene translation (Fig. 5). Inactivation of CDK12 leads to focal tandem duplications that increase gene fusions or mutations, thus enhancing neoantigen production and the tumor immune response (Fig. 5).^351^ Two of four mCRPC patients with CDK12 mutations had obvious PSA responses after administration of a PD1 inhibitor.^351^ A phase 2 clinical trial to evaluate the efficacy of ipilimumab and nivolumab for CDK12-mutated mCRPC is ongoing (Table 6).

## Targeting the cell cycle

### CDK4/6 and cell cycle

Hyperproliferation is a hallmark of cancer development. The cell cycle can be divided into four ordered phases: G1 (gap 1), S (DNA synthesis), G2 (gap 2), and M (mitosis), which are precisely controlled by molecules such as CDKs. The key regulatory checkpoints in the G1 and G2 phases determine whether cells enter the S phase and mitosis. CDK4 and CDK6, two serine/threonine kinases, are crucial for governing the transition from the G1 to S phase.^352^ CDK4/6 is activated by the binding of cyclin D1/2/3 during the early G1 phase in response to mitogenic stimuli. The cyclin D-CDK4/6 complexes subsequently phosphorylate and inactivate the retinoblastoma (RB) tumor suppressor protein.^353,354^ RB proteins typically bind to the transcription factor E2Fs, such as E2F1, to limit the expression of many E2F target genes that are involved in the cell cycle and mitotic progression.^355,356^ Phosphorylation by CDK4/6 reduces the binding affinity of RB to E2F, leading to transactivation of E2F transcription factors such as E2F1 (Fig. 1c). Activated E2F1 recruits RNA-POLII to induce the transcription of CDK2, E-type cyclins, and other cell cycle-related proteins that further phosphorylate RB and promote the G1-to-S-phase cell cycle transition.^357,358^ CDK4/6 also phosphorylates other substrates and plays an important role in differentiation and metabolism.^359,360^

### CDK4/6 inhibitors

Currently, three new CDK4/6 inhibitors, palbociclib (PD0332991), ribociclib (LEE011), and abemaciclib (LY2835219), have entered early phase trials for PCa (Table 7). A recent phase 2 clinic study evaluated the efficacy of ADT (including bicalutamide, goserelin, and leuprolide) plus palbociclib in patients with RB-positive metastatic hormone-sensitive PCa. The primary PSA endpoint was met in 80% of patients in both the ADT alone and ADT plus palbociclib groups (16/20 versus 32/40; *p* = 0.87), while 1-year biochemical progression-free survival (PFS) was 69% in the ADT alone group and 74% in the ADT plus palbociclib group.^361^ Although these important clinical data are still not sufficient, this study suggests that co-targeting of AR signaling and the cell cycle is possible. Furthermore, abiraterone plus abemaciclib was evaluated in a phase 2/3 trial, and ribociclib plus enzalutamide or docetaxel are under investigation in different trials for the treatment of mCRPC (Table 7). PCa has shown limited response to immunotherapy because of its cold tumor environment.^348^ Notably, CDK4/6 inhibitors have been shown to increase the tumor immune response and TILs,^362,363^ which supports the potential synergistic effects of CDK4/6 inhibitors and ICIs. Additional studies are trying to identify the population of appropriate patients and synergistic combinations that would make these agents more efficacious.

**Table 7:** Selective clinical trials of CDK4/6 inhibitors or p53-targeted agents

Drug	Target	Condition	Status	Phase	NCT identifier
Palbociclib (PD0332991)	CDK4/6	RB-positive metastatic PCa	Completed	2	NCT02059213
Palbociclib (PD0332991)	CDK4/6	mCRPC	Not yet recruiting	2	NCT02905318
Ribociclib (LEE011)	CDK4/6	mCRPC	Completed	2	NCT02494921
Ribociclib (LEE011)	CDK4/6	RB-positive metastatic PCa	Recruiting	2	NCT02555189
Abemaciclib (LY2835219)	CDK4/6	mCRPC	Recruiting	2/3	NCT03706365
Abemaciclib (LY2835219)	CDK4/6	Locally advanced PCa	Recruiting	2	NCT04298983
Abemaciclib (LY2835219)	CDK4/6	mCRPC	Not yet recruiting	2	NCT04408924
APR-246	Mutant p53	Refractory PCa	Completed	1	NCT00900614
Arsenic trioxide	Mutant p53	Stage IV PCa	Completed	2	NCT00004149

### p53 and the targeting approaches

The tumor suppressor p53 is widely known as the “genome guardian.” Activated p53 binds to a specific DNA sequence as a tetramer to promote gene expression (such as *CDKN1A*, *BAX*, *PUMA*, and *NOXA*), thus inducing cell apoptosis and cell-cycle arrest.^364^ The *TP53* gene, which encodes the p53 protein, is frequently mutated in PCa, especially in neuroendocrine-like mCRPC. The combined alteration of *RB* and *TP53* occurs in 74% of neuroendocrine-like mCRPC.^365^ p53 mutations are predominantly distributed throughout the DBD, and these p53 mutations either lose their DNA-binding ability or form a heterodimer complex with wild-type p53 to attenuate wild-type p53 functions, thus disrupting the tumor-suppressive functions of p53.^366^ Moreover, many mutant p53 proteins acquire gain-of-function activities, which enable them to deactivate the other p53 family members, specifically p73 and p63.^367^ Under normal conditions, p53 has a half-life of less than 20 min owing to the feedback regulation of proteasome-mediated p53 degradation by E3 ubiquitin-protein ligase MDM2.^368^

p53 alteration fosters more lethal PCa; thus, targeting p53 is an attractive therapeutic strategy for aggressive PCa. Approaches for targeting p53 can be summarized as follows: first, compounds such as idasanutlin (RG7388) and RG7112 were developed to prevent degradation of wild-type p53 by blocking p53-MDM2 interactions,^369,370^ thus maintaining their ability to suppress tumor formation. Although no clinical studies exist for PCa, multiple p53-MDM2 antagonists are undergoing clinical trials; of these, idasanutlin is the most advanced and is under testing in a phase 3 clinical trial in patients with refractory acute myeloid leukemia.^371^ Second, pharmacological reactivation of mutant p53 uses small molecules like APR-246, COTI-2, and arsenic trioxide (Table 7), which bind to mutant p53 and convert the protein to a p53 wild-type-like conformation, thus restoring wild-type DNA-binding properties.^372–374^ Based on the results of a phase 1b/2 trial for myelodysplastic syndrome,^375^ the FDA granted Fast Track designation to APR-246 for the treatment of myelodysplastic syndrome in patients with a p53 mutation.^376^ APR-246 was tested in PCa, and showed a favorable pharmacokinetic profile in a phase 1 trial (Table 7).^377^ Third, mutant p53 neoantigens can elicit intratumoral T-cell responses;^378^ therefore, p53 neoantigens are considered to be promising targets. For example, a recent study generated a T-cell-based therapy that links T cells to cancer cells using a novel antibody that specifically binds to the p53^R175H^ peptide-MHC complex, lysing cancer cells depending on the presence of the neoantigen.^379^ Clinical studies targeting mutant p53 neoantigens in PCa are rare. More clinical studies on p53-targeted agents in PCa are warranted, and it is projected that at least several of these molecules will prove effective in the future.

## Targeting the PI3K/AKT/mTOR signaling axis

### PTEN/PI3K/AKT/mTOR signaling

Inactivation of PTEN (phosphatase and tensin homologue) by deletion or mutation has been identified in ~20% of primary PCa and approximately 35% of CPRC cases (Fig. 1a).^380^ PTEN, a dual-specificity phosphatase, converts phosphatidylinositol-3,4,5-trisphosphate (PIP_3_) into phosphatidylinositol 4,5-bisphosphate (PIP_2_),^381^ causing PTEN to function as a direct antagonist of the activity of class I PI3K, which converts PIP_2_ to PIP_3_. As a result, PTEN loss leads to aberrant accumulation of PIP3 on cell membranes, which leads to the recruitment of PDK1 to phosphorylate its substrate AKT. Phosphorylated AKT subsequently regulates several downstream signaling cascades, including mTOR, which is crucial for protein synthesis, autophagy, cellular proliferation, and metabolism.^382^ PTEN acts as a gatekeeper of the PI3K/AKT/mTOR pathway. Therefore, PTEN deletions or mutations are strongly associated with activation of PI3K/AKT/mTOR signaling and poor prognosis in advanced PCa.^380^

### PI3K, ATK, mTOR, and PIM (proviral-integration site for Moloney-murine leukemia virus) inhibitors

PI3K, a plasma membrane-associated protein kinase, is formed by two functional subunits: a catalytic subunit (p110α, p110β, or p110δ) and a regulatory subunit (p85α, p55α, p50α, p85β, or p55γ isoform).^383^ The catalytic subunit p110β is believed to be the most relevant isoform for PCa progression.^384,385^ PI3K inhibitors, such as BKM120 and PX866, target the catalytic subunits of all three isoforms (p110α, p110β, and p110δ). BKM120 showed a partial response in a phase 1 study.^386^ PX866, a derivative of wortmannin, was well tolerated in patients with recurrent mCRPC.^387,388^ However, monotherapy with PI3K inhibitors is limited in the clinic.

AKT, a serine/threonine protein kinase, is the main downstream effector of PI3K, and is fully activated when both Thr308 and Ser473 sites are phosphorylated.^389^ Activated AKT phosphorylates several targets, such as mTOR, GSK3, FOXO, and AMPK, which are involved in multiple cellular processes.^390,391^ Thus far, the AKT inhibitors that have reached the clinical phase include allosteric inhibitors (such as perifosine and MK-2206) and ATP-competitive inhibitors (such as capivasertib, ipatasertib, and uprosertib). Of note, the combination of capivasertib with docetaxel resulted in a greater than 50% reduction in PSA levels in 70% of men with mCRPC in a phase 1 study.^392^ Ipatasertib prolonged PSA progression-free interval and overall survival compared to the placebo group in a phase 2 trial.^49^ A phase 3 trial to evaluate the efficacy of abiraterone plus ipatasertib for the treatment of mCRPC is ongoing (Table 8).

**Table 8:** Selective clinical trials of PI3K, AKT and mTOR inhibitors

Drug	Target	Condition	Status	Phase	NCT identifier
Buparlisib (BKM120)	PI3K	CRPC	Completed	1	NCT01634061
Buparlisib (BKM120)	PI3K	CRPC	Terminated	2	NCT01385293
Dactolisib (BEZ235)	PI3K	mCRPC	Terminated	1/2	NCT01717898
Samotolisib (LY3023414)	PI3K	CRPC	Completed	2	NCT02407054
AZD8186	PI3K	CRPC	Completed	1	NCT01884285
GSK2636771	PI3K	CRPC	Completed	1	NCT02215096
Perifosine (KRX-0401)	AKT	mCRPC	Completed	2	NCT00060437
MK2206	AKT	Advanced cancers	Completed	1	NCT01295632
Ipatasertib (GDC-0068)	AKT	Locally advanced PCa	Recruiting	1/2	NCT04737109
Ipatasertib (GDC-0068)	AKT	mCRPC	Not yet recruiting	3	NCT03072238
Capivasertib (AZD5363)	AKT	CRPC	Completed	1	NCT04087174
Sirolimus	mTOR	Locally advanced PCa	Completed	1/2	NCT00311623
Ridaforolimus (MK8669)	mTOR	mCRPC	Completed	2	NCT00777959
Temsirolimus (CCI-779)	mTOR	CRPC	Completed	2	NCT00919035
Everolimus (RAD001)	mTOR	CRPC	Completed	2	NCT00814788
Sapanisertib (MLN0128)	mTOR	mCRPC	Completed	2	NCT02091531
Vistusertib (AZD2014)	mTOR	PCa	Completed	1	NCT02064608
Apitolisib (GDC-0980)	PI3K, mTOR	CRPC	Not yet recruiting	1/2	NCT01485861
CC-115	mTOR, DNA-PK	CRPC	Not yet recruiting	1	NCT02833883

The serine/threonine protein kinase mTOR, the major downstream effector of AKT signaling, interacts with different proteins and forms two distinct complexes, mTORC1 and mTORC2.^393^ Several types of mTOR inhibitors exist, including mTORC1 inhibitors (such as rapamycin, everolimus, and temsirolimus), mTORC1/2 dual inhibitors (such as sapanisertib and vistusertib), and dual PI3K-mTORC1/2 inhibitors (such as apitolisib and BEZ235). Clinical trials using single mTORC1 inhibitors showed predictable toxicity with no favorable clinical responses in mCRPC.^394,395^ Sapanisertib was previously tested in a phase 2 study in advanced CRPC but showed limited clinical efficacy.^47^ Vistusertib was tested in men with high-risk PCa and was administered prior to radical prostatectomy in a phase 1 trial (Table 8). Currently, apitolisib plus abiraterone is being tested for CRPC in phase 1/2 clinical trials (Table 8). A novel mTOR and DNA-PK (DNA-dependent protein kinase) dual inhibitor, CC-155, was evaluated in a phase 1 study (Table 8).

PIM kinases have been found to sustain the PI3K/AKT/mTOR pathway.^396,397^ Increased expression of PIM family members has been detected in PCa, and PIM confers resistance not only to PI3K/AKT inhibitors, but also to chemotherapy and radiotherapy.^398^ Therefore, co-targeting PIM and PI3K/AKT/mTOR could offer superior clinical outcomes relative to targeting either of these alone. The combination of the PIM inhibitor AZD1208 and the PI3K/mTOR inhibitor BEZ235 (dactolisib) has been investigated in clinical trials.^399^ A novel and highly efficient triple PIM/PI3K/mTOR inhibitor AUM302 elicited a superior functional outcome compared to the effects of the combination of AZD1208 and BEZ235; these results may help reduce treatment toxicity in future trials.^399^ Overall, the clinical application of PI3K/AKT/mTOR inhibitors is still limited in PCa, and further studies that can identify new biomarkers for patient selection or improve co-targeting strategies are still required to enhance their therapeutic effects.

## Targeting epigenetic marks

### Epigenetic modifications

**Fig. 6:**
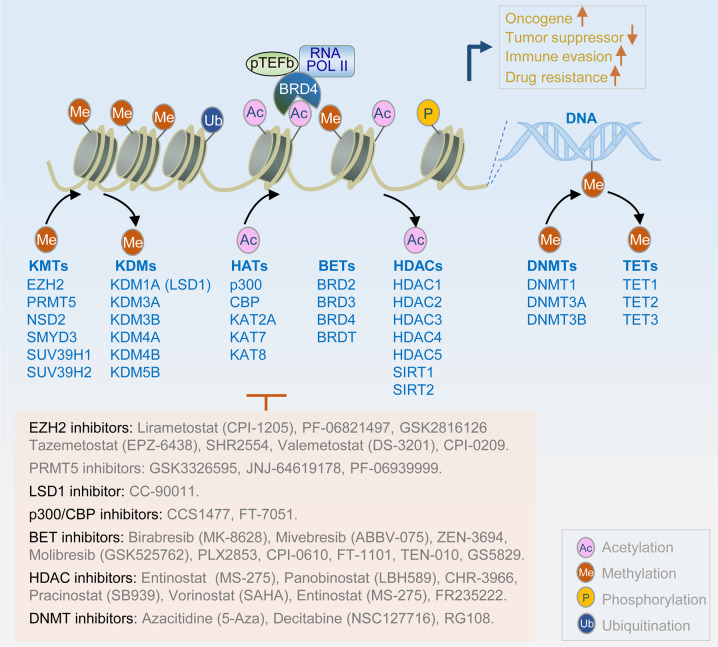
Schematic of major histone or DNA modification and the key modifiers implicated in PCa. Aberrant histone (such as acetylation, methylation, phosphorylation, and ubiquitination) or DNA modifications (such as methylation) might upregulate oncogenes or reduce tumor suppression genes; thus, targeting these epigenetic modifications is an attractive strategy to treat PCa. Several compounds (such as EZH2 inhibitors, LSD1 inhibitor, BET inhibitors, HDAC inhibitors and DNMT inhibitors) based on epigenetic targets have entered clinical trials in succession

Epigenetic traits are heritable phenotypes attributable to changes in chromosomes or DNA modifications without alterations in the DNA sequence.^400^ In addition to genomic changes, epigenetic alterations (such as histone modifications and DNA methylation) have been reported to be associated with PCa progression.^401–403^ Epigenetic modifications, including acetylation, methylation, ubiquitination, and phosphorylation, play critical roles in transcription, DNA repair, and replication.^404^ Epigenetic regulation is a dynamic and reversible process that adds epigenetic marks onto either histones or DNA by epigenetic writers, recognizes or recruits epigenetic marks by epigenetic readers, and removes epigenetic marks by epigenetic erasers (Fig. 6). Aberrant histone modifications may upregulate oncogenes or reduce the expression of tumor suppressor genes. Importantly, histone methylation/acetylation and DNA methylation play a central role in controlling gene expression, thus promoting the progression and metastasis of PCa.^405,406^

### Histone methylation

Histones are methylated by the addition of one, two, or three methyl groups from S-adenosylmethionine to the side chains of the arginine, lysine, and histidine residues. Histone methylations such as H3K4me1, H3K9me2, and H3K9me3 have been reported to be reduced in PCa tissues compared to those in non-malignant tissues;^407^ however, in comparison with localized PCa and normal prostate tissues, H3K27me3 marks at promoter regions of tumor suppressor genes were strongly enriched in metastatic PCa.^403,408^ Overexpression of EZH2, a histone methyltransferase, is a major reason for the increased genomic distribution of H3K27me3 in metastatic PCa.^409^ EZH2 plays an important role in promoting lineage plasticity and differentiation changes, which are highly associated with NEPC.^410^ Thus, EZH2 is an attractive target, and many EZH2 inhibitors (lirametostat, tazemetostat, valemetostat, PF-06821497, and SHR2554) have emerged in early clinical studies (Table 9). The effectiveness of EZH2 inhibitors alone, in combination with AR inhibitors, or in combination with immunotherapy for the treatment of PCa is currently under evaluation in clinical trials (Table 9).

**Table 9:** Selective clinical trials of epigenetic inhibitors

Drug	Target	Condition	Status	Phase	NCT identifier
Lirametostat (CPI-1205)	EZH2	mCRPC	Not yet recruiting	1/2	NCT03480646
PF-06821497	EZH2	CRPC	Recruiting	1	NCT03460977
Tazemetostat (EPZ-6438)	EZH2	mCRPC	Recruiting	1	NCT04846478
Tazemetostat (EPZ-6438)	EZH2	mCRPC	Recruiting	1/2	NCT04179864
SHR2554	EZH2	CRPC	Terminated	1/2	NCT03741712
Valemetostat (DS-3201)	EZH2	mCRPC	Recruiting	1	NCT04388852
CC-90011	LSD1	mCRPC	Recruiting	1	NCT04628988
CCS1477	p300/CBP	mCRPC	Recruiting	1/2	NCT03568656
FT-7051	p300/CBP	mCRPC	Recruiting	1	NCT04575766
Birabresib (MK-8628)	BET bromodomain	CRPC	Completed	1	NCT02259114
Mivebresib (ABBV-075)	BET bromodomain	PCa	Completed	1	NCT02391480
Molibresib (GSK525762)	BET bromodomain	CRPC	Completed	1	NCT03150056
ZEN-3694	BET bromodomain	mCRPC	Completed	1/2	NCT02711956
ZEN-3694	BET bromodomain	mCRPC	Recruiting	2	NCT04471974
PLX2853	BET bromodomain	mCRPC	Not yet recruiting	1/2	NCT04556617
Entinostat (MS-275)	HDACs	CRPC	Terminated	1	NCT03829930
Panobinostat (LBH589)	HDACs	CRPC	Completed	1	NCT00878436
Pracinostat (SB939)	HDACs	CRPC	Completed	2	NCT01075308
Vorinostat (SAHA)	HDACs	Metastatic PCa	Completed	2	NCT00330161
Azacitidine (5-Aza)	DNMT	PCa	Completed	2	NCT00384839
Decitabine (NSC127716)	DNMT	PCa	Withdrawn	1/2	NCT03709550

In contrast, histone demethylase catalyzes the removal of methyl groups from histones. Multiple histone demethylases, such as LSD1 (also known as KDM1A), are overexpressed in patients with advanced PCa.^411^ LSD1 demethylates H3K4me1 and H3K4me2.^412^ LSD1 co-operates with AR and activates AR-dependent transcription or a subset of cell-cycle gene expression.^413,414^ A clinical trial with a novel LSD1 inhibitor CC-90011 was recently launched (Table 9).^415^

### Histone acetylation

Histone acetylation is achieved by the addition of an acetyl group to the lysine residues of histones, and is linked to open and active chromatin. Histone acetylation is usually correlated with activating transcription, whereas histone deacetylation is commonly associated with gene silencing.^416^ Super-enhancers, a cluster of enhancers marked by high H3K27ac levels, play a key role as an oncogenic driver in cancer cells.^417,418^ Activation of histone acetyltransferases, such as p300 and CBP (CREB-binding protein), is highly associated with increased modification of H3K27ac.^419^ Furthermore, p300 and CBP play crucial roles in regulating key genes in PCa, including AR target genes.^420^ Recently, p300 and CBP inhibitors (such as CCS1477, A-485, and FT-7051) have been developed.^421,422^ Clinical trials to test the efficacy of CCS1477 and FT-7051 in PCa have recently begun (Table 9).

In contrast, HDACs can remove acetylation of histones. Eighteen different types of HDACs have been identified in humans,^423^ and overexpression of HDACs occurs in different malignancies, including PCa.^424^ HDAC1 and HDAC2 expression is positively correlated with higher Gleason scores of PCa, while the expressions of HDAC1, HDAC2, and HDAC3 are positively associated with the proliferative marker Ki67.^425^ HDAC inhibitors are potential therapeutic options because the expression of HDACs is associated with poor clinical outcomes.^425^ There are five classes of HDAC inhibitors, including hydroxamic acids, cyclic tetrapeptides, short chain carboxylic acids, benzamides, and keto-derivatives.^426^ Several HDAC inhibitors, including vorinostat, pracinostat, panobinostat, and romidepsin, have been tested in phase 2 clinical trials for PCa (Table 9); however, most patients exhibited either toxicity from these agents or disease progression.^427^ Clinical trials involving HDAC inhibitors have not achieved significant success because of poor oral bioavailability, non-selectivity of the drugs, or other mechanisms that remain to be explored.^427^

### BET protein

Acetylated lysines are recognized by a class of proteins containing bromodomains, such as the BET proteins BRD2, BRD3, BRD4, and BRDT.^428^ Acetylated lysine residues in histones can be bound by BET proteins via BD1 and BD2 bromodomains, which is the key step in regulating transcription.^428^ Importantly, the expression of BRD4 is significantly associated with poor clinical outcomes.^429,430^ BET proteins, such as BRD4, are tightly linked to AR signaling activation and drug resistance in SPOP mutant PCa.^431–433^ Numerous BET inhibitors, including pan-BET bromodomain inhibitors (such as JQ1, I-BET151, birabresib, mivebresib, and ZEN-3694) and selective inhibitors (such as ABBV-744, molibresib, and PLX2853), have been demonstrated to have antitumor effects in preclinical models. Birabresib (MK-8628) and mivebresib (ABBV-075) were tested in patients with solid tumors, including CRPC, but neither birabresib nor mivebresib demonstrated significant antitumor activity in CRPC patients (Table 9).^59,61^ However, a clinical trial at phase 1/2 demonstrated that enzalutamide plus ZEN-3694 prolonged the PFS in a subset of patients with mCRPC resistant to enzalutamide and/or abiraterone.^62^ A new study found molibresib (GSK525762) was well tolerated in patients.^434^ Recently, phase 1 or phase 2 clinical trials of ZEN-3694 and PLX2853, in combination with AR inhibitors (enzalutamide or abiraterone) have been launched in CRPC patients (Table 9). Ongoing clinical studies of BET inhibitors are needed to demonstrate their safety profiles and the role of pharmacodynamic regulation of AR signaling in patients.

### DNA methylation

DNA methylation is achieved when a methyl group is added to the C5 position of cytosine residues in the CpG dinucleotides, and is linked to gene silencing.^435^ DNMT enzymes catalyze 5-methyl cytosine (5mC) in DNA, whereas these marks can be removed by the DNA demethylases TET (ten-eleven translocation) family.^436^ Approximately 60% of all promoters colocalize with CpG islands^437^; therefore, aberrant DNA hypermethylation at CpG islands can lead to gene silencing, such as inactivation of tumor suppressor genes.^88,438^ A previous studied showed that 22% of tumors were associated with hypermethylation in PCa.^439^ DNMT inhibitors azacytidine (5-Aza) and decitabine have been developed to target aberrant DNA hypermethylation. Azacitidine and decitabine (NSC127716) were clinically evaluated for PCa (Table 9). A phase 1/2 study of azacitidine in combination with docetaxel in mCRPC demonstrated a PSA response in 52% (10/19) of patients, without exhibiting dose-limiting toxicity.^57^

## Targeting WNT signaling

### WNT/β-catenin signaling

**Fig. 7:**
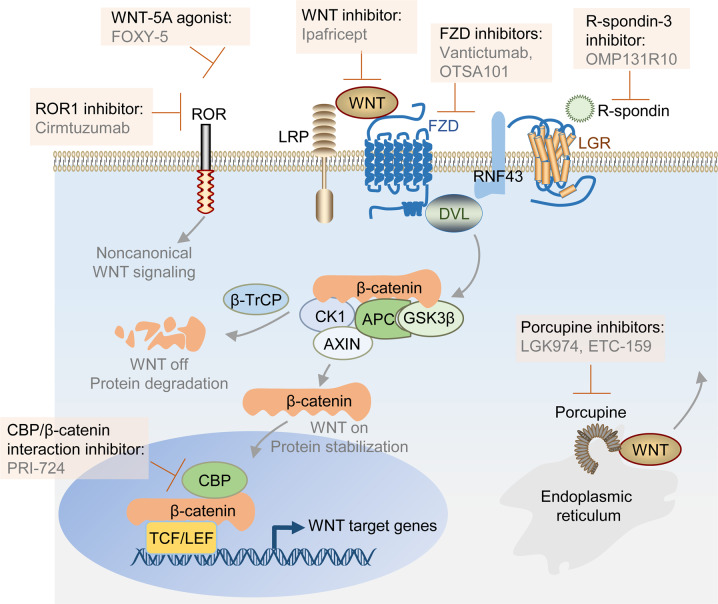
The WNT signaling pathway and targeting therapeutic strategies. WNT ligands bind to FZD and LRP5/6 receptors to phosphorylate DVL, and then phosphorylated DVL recruits the destruction complex to the plasma membrane. This inhibits GSK3 and prevents phosphorylation of β-catenin, resulting in the stabilization and accumulation of β-catenin proteins to form a complex with TCF/LEF in the nucleus, thereby activating the transcription of downstream target genes. There are several targeting strategies that prevent the activation of WNT signaling, such as targeting WNT ligands and their receptors, inhibiting the WNT secretion by targeting porcupine, and disrupting the interaction between CBP and β-catenin

The canonical WNT/β-catenin pathway is activated in late-stage PCa, and promotes tumor cell growth and drug resistance in PCa.^440^ The binding of WNT ligands to their receptors in cell surface activates signaling pathways that regulate cell differentiation and proliferation.^441^ In the absence of WNT ligands, cytoplasmic β-catenin is rapidly degraded by a destruction complex, whose components contain adenomatous polyposis coli protein (APC), AXIN, casein kinase 1 (CK1), β-transducin-repeat-containing protein (β-TrCP), and glycogen synthase kinase 3 (GSK3) (Fig. 7).^65,441,442^ When WNT ligands bind to frizzled (FZD) receptors and co-receptors LRP5/6, LRP5/6 are phosphorylated by CK1 and GSK3, and then the signal is transduced to activate the cytoplasmic phosphoprotein dishevelled (DVL). Phosphorylated DVL recruits the destruction complex to the plasma membrane. This inhibits GSK3 and prevents phosphorylation of β-catenin, thereby resulting in the stabilization and accumulation of β-catenin proteins. Sequentially, β-catenin proteins translocate into nucleus, form a complex with T-cell factors (TCFs)/lymphoid enhancer-binding factor 1 (LEF1), recruit transcription factors and co-activators, such as the CBP/p300, and activate the transcription of downstream target genes, including *ABCB1*, *MYC*, *MYCN*, *NEUROG1*, *NEUROD1*, *SOX2*, *SUZ12*, *TWIST*, and *YAP* (Fig. 7).^65,441–443^ Importantly, activating mutations of the WNT/β-catenin pathway genes were enriched in metastatic PCa (19%) compared to those in primary PCa (6%).^444^ An increasing number of studies have indicated that the activation of WNT/β-catenin signaling is highly linked to cell proliferation, invasion, bone metastasis, drug resistance, and neuroendocrine differentiation in the late stage of PCa.^445–455^

### WNT signaling inhibitors

Numerous agents that target different components of the WNT pathway have been developed and tested in clinical trials (Fig. 7), although only a few have been associated with PCa. Several strategies can prevent the activation of WNT signaling. First, targeting WNT ligands and their receptors with monoclonal antibodies and small molecules is an attractive therapeutic strategy. The WNT ligand WNT-5A was targeted using a WNT-5A-mimicking peptide named Foxy-5.^456^ Foxy-5 was evaluated in a phase 1 clinical trial in patients with solid malignant tumors including PCa (Table 10). An R-spondin-3 antibody (OMP-131R10) was tested in a phase 1 clinical trial for advanced solid tumors.^65^ The antibody OMP-18R5 (vantictumab) targets FZD1, FZD2, FZD5, FZD7, and FZD8.^65^ OTSA101 is a radiolabeled antibody against FZD10.^457^ Pafricept (OMP-54F28) is a decoy WNT receptor that has been tested in a phase 1a clinical trial and has demonstrated evidence of WNT pathway inhibition,^458^ but its development has been terminated due to bone-related toxicity.^459^ Second, inhibition of WNT secretion by targeting porcupine, a membrane-bound O-acetyltransferase, is another optional strategy. Porcupine is important for WNT palmitoylation, which is essential for the secretion of WNT proteins.^460^ Several porcupine inhibitors, such as IWP-2, WNT-C59, LGK974 (WNT974), and ETC-159, also have been developed^65,461^, and of these, LGK974 (discontinued) and ETC-159 (phase 1) have entered trials in patients with advanced solid tumors.^462^ Third, the interaction between cofactor CBP and β-catenin is critical for transcriptional activation of β-catenin.^65^ Thus, disruption of the interaction between CBP and β-catenin provides a new strategy to prevent WNT signaling. ICG-001 and its derivative PRI-724 were found to inhibit the binding of β-catenin to CBP.^463^ PRI-724, which has an acceptable toxicity profile, was tested in phase 2 clinical trials for metastatic colorectal cancer (but was withdrawn owing to drug supply issues) and advanced myeloid malignancies (completed).^463^

**Table 10:** Selective clinical trials of other signaling pathway inhibitors

Drug	Target	Condition	Status	Phase	NCT identifier
FOXY-5	WNT-5A receptors	PCa	Completed	1	NCT02020291
Bevacizumab	VEGF-A	PCa	Completed	3	NCT00110214
Zibotentan (ZD4054)	ETAR	mCRPC	Completed	3	NCT00554229
Erdafitinib (JNJ-42756493)	FGFR	DN-PCa	Suspended	2	NCT03999515
Cetuximab	EGFR	mCRPC	Completed	2	NCT00728663
Gefitinib (ZD1839)	EGFR	PCa	Completed	2	NCT00265070
Dovitinib (TKI258)	FGFR, VEGFR, PDGFR	CRPC	Completed	2	NCT01741116
Sunitinib (SU11248)	VEGFR, PDGFR	mCRPC	Terminated	3	NCT00676650
Cabozantinib (XL184)	VEGFR, c-MET, c-KIT	mCRPC	Completed	3	NCT01605227
Masitinib (AB1010)	KIT, PDGFR, FGFR	mCRPC	Completed	3	NCT03761225
Galunisertib (LY2157299)	TGFβR1	mCRPC	Recruiting	2	NCT02452008
M7824	TGFβ, PD-L1	Metastatic PCa	Recruiting	1/2	NCT04633252
Dasatinib (BMS-354825)	SRC, c-KIT	CRPC	Completed	3	NCT00744497
Saracatinib (AZD0530)	SRC	CRPC	Completed	2	NCT00513071
Trametinib (GSK1120212)	MEK	mCRPC	Not yet recruiting	2	NCT02881242

Although targeting WNT signaling is very attractive for treating late-stage PCa, WNT signaling inhibitors are in early stages of development and application, and several limitations remain. First, WNT signaling is complicated because there are 19 WNT-secreted glycoproteins and more than 15 types of WNT receptors in humans,^464,465^ which activate different downstream pathways. Second, variations in and balances between canonical and noncanonical WNT signaling is elusive,^466^ making it even more difficult to target the WNT pathway. Third, WNT signaling plays a fundamental role in the homeostasis of the intestine, hair follicles, and hematopoietic system;^464^ thus, blocking the WNT pathway could cause systemic toxicity. Prospective studies include: (I) the combination of WNT inhibitors with other cancer drugs or immunotherapies to limit toxicities or improve therapeutic efficacy, (II) the classification of disease subtypes to distinguish methods of WNT activation among different stages of disease, and (III) the expansion of models to more clearly understand mechanism of action between the canonical and noncanonical WNT pathways and thus, discover novel therapeutic approaches.

## Targeting other pathways

### VEGF

VEGF is a prominent factor involved in angiogenesis and is highly associated with tumor growth, including in PCa.^66,467^ Angiogenesis is essential for tumor growth, because the newly formed blood vessels are important to sustain adequate energy and oxygen.^468,469^ The binding of VEGFs (VEGFA, VEGFB, VEGFC, and VEGFD) to cell surface receptors (VEGFR1, VEGFR2, VEGFR3) activates signaling pathways that play important roles in cell growth and motility in PCa.^468^ Of note, VEGFA is the founding member of the vascular permeability factor, and is frequently overexpressed in PCa.^470,471^ Overexpression of VEGFA in PCa is associated with angiogenesis, recurrence, and advanced disease stages among patients.^472,473^ Importantly, bevacizumab, a humanized monoclonal antibody against VEGFA, is used to treat several different cancers.^474^ A phase 3 clinical study in mCRPC patients demonstrated that the combination of bevacizumab and docetaxel did not result in a significant increase in median overall survival versus docetaxel alone (22.6 versus 21.5 months; *p* = 0.181); however, improvements were found in median PFS (9.9 versus 7.5 months; *p* < 0.001) and major PSA response (69.5% versus 57.9%; *P* < 0.001).^475^

### ETAR

Activation of ETAR by endothelin-1 is involved in tumor progression through angiogenesis, invasion, apoptosis, and the effect of the bone microenvironment.^476,477^ Activated ETAR signaling promotes osteoblast proliferation and new bone formation, which is highly associated with bone metastasis in PCa.^478,479^ Thus, the ETAR inhibitors zibotentan (ZD4054) and atrasentan have been tested in men with mCRPC. In a phase 2 trial, zibotentan prolonged overall survival from 17.3 to 24.5 months in patients with mCRPC,^480^ but a phase 3 trial did not result in a statistically significant improvement in overall survival in the patients.^481^ Similarly, atrasentan delayed PFS and PSA progression in a phase 2 trial,^482^ but a phase 3 trial demonstrated that atrasentan did not significantly reduce the disease progression time in CRPC patients.^483^ However, a phase 3 trial in mCRPC demonstrated that patients with the highest level of bone metabolism markers (such as pyridinoline and alkaline phosphatase) obtained a survival benefit from atrasentan compared to that from placebo (13 versus 5 months; *p* = 0.005).^484^

### TGFβ

TGF-β is a cytokine that regulates many cellular functions, such as cell differentiation and migration. Binding of TGFβ to TGFβR2 (TGFβ receptor 2) phosphorylates and activates TGFβR1 (TGFβ receptor 1). Subsequently, activated TGFβR1 phosphorylates SMAD2 and SMAD3 proteins, leading to SMAD2/SMAD3 complexes with SMAD4. Next, these complexes are translocated into the nucleus and stimulate target gene expression.^485,486^ Increased TGFβ or its target genes in PCa are associated with a more aggressive disease, metastasis, and poor prognosis.^487–489^. TGFβ also plays an important role in the context of the bone microenvironment and supports the progression of bone metastasis in PCa.^490–492^ Furthermore, TGFβ facilitates tumor growth through its immunosuppressive function.^493,494^ To target TGFβ signaling, galunisertib (LY2157299), an oral small-molecule inhibitor of TGFβR1, has been developed.^495^ A clinical study of galunisertib plus enzalutamide in mCRPC has been initiated (Table 10). Furthermore, a clinical trial of M7824,^496^ targeting both TGFβ and PD-L1, also has been launched for the treatment of metastatic PCa (Table 10).

### RTKs and nonreceptor tyrosine kinases (NRTKs)

Both RTKs (such as FGFR, EGFR, PDGFR, and VEGFR) and NRTKs (such as SRC) are very important in the carcinogenesis and progression of PCa, and are potential targets for the treatment of PCa.^497,498^ Signal transductions through ligands binding with RTKs or stimulation of unique NRTKs lead to cross-phosphorylation of specific tyrosine residues, which activate downstream signaling such as PI3K/AKT, phospholipase C, and Janus tyrosine kinase.^499,500^ The signaling subsequently regulates the transcription of genes involved in proliferation, survival and differentiation.^499^

Fibroblast growth factor (FGF) receptors (FGFR1/4) and their ligands (FGF1/2/4/8/17) are overexpressed in PCa.^501–504^ Enhanced FGF signaling leads to tumor progression, angiogenesis, epithelial-to-mesenchymal transition (EMT), and upregulation of AR.^505–507^ Moreover, FGF/FGFR activation is highly correlated with AR-null PCa and drug resistance.^508^ Therefore, inhibition of the FGF axis may be a viable strategy for the treatment of PCa. Erdafitinib (JNJ-42756493),^509^ a small oral molecule that works against all four FGFR family members, is being tested in a phase 2 clinical study (Table 10) of AR-null and neuroendocrine-null PCa, termed double-negative prostate cancer (DNPC).

EGFR mutation or overexpression leads to malignant progression in many cancer types.^510^ In PCa, increased expression of EGFR correlates with a high Gleason score and advanced stage disease,^511^ and activation of EGFR promotes metastatic progression and recurrence.^512^ EGFR inhibitors such as gefitinib (a small-molecule compound) and cetuximab (a monoclonal antibody) have been widely used in metastatic colorectal cancer and non-small cell lung cancer. In a phase 2 clinical trial, gefitinib did not exhibit any response to PSA or objective measurable disease in patients with CRPC.^510^ However, in another trial, cetuximab resulted in an obvious PSA decline in many patients, and improved PFS was found in patients with EGFR overexpression.^513^

The SRC signaling pathway has many effects on tumorigenesis and tumor progression.^73,498^ In PCa, SRC activity is highly increased in bone metastasis.^73,514–516^ Encouraging results in preclinical studies of PCa led to several clinical studies of SRC inhibitors, such as dasatinib (BMS-354825)^517,518^ and saracatinib (AZD0530).^519,520^ However, a phase 3 study of docetaxel plus dasatinib did not improve the overall survival in patients with mCRPC, despite the fact that the PSA progression time was delayed.^518^ Similar results were found in the trials of abiraterone plus dasatinib.^517,521^ A phase 2 study of saracatinib also failed to achieve satisfactory oncological outcomes, while adverse events were observed, such as dehydration, thrombocytopenia, and weakness.^519^

Moreover, multitargeted tyrosine kinase inhibitors, such as dovitinib (TKI258),^522^ sunitinib (SU11248),^523,524^ cabozantinib (XL184),^525^ and masitinib (AB1010)^526^, have entered clinical trials for PCa (Table 10). For example, dovitinib, an oral multitargeted RTK inhibitor, potently inhibits FGFR1/2/3, VEGFR1/2/3, PDGFR, and KIT.^522^ A phase 2 clinical study found that dovitinib showed a better response in chemotherapy-naive patients and patients with high serum VEGFR2.^522^ Finally, cabozantinib,^525^ a small-molecule inhibitor of c-MET and VEGFR2, has been evaluated in two phase 3 clinical trials, which reported no survival benefit of cabozantinib, suggesting that further rational development may be justified.^527–529^

### MEK

Aberrant activation of mitogen-activated protein kinase (MAPK) signaling resulting from upstream activating mutations, such as the rat sarcoma viral oncogene homolog (RAS), v-raf-1 murine leukemia viral oncogene homolog (RAF), and growth factors, converges with MEK. Increased activation of the RAS/RAF/MEK/extracellular signal-regulated kinase (ERK) pathway has been associated with poor prognosis and androgen independence in advanced PCa.^508,530,531^ Increased expression of MAPK pathway members and high levels of phosphorylated ERK1/2 were observed in mCRPCs.^532–535^ Pharmacological targeting of the MEK/ERK pathway may be a viable strategy for patients with mCRPC, and the MEK1/2 inhibitor trametinib (GSK1120212) is currently being tested in a phase 2 trial of patients with mCRPC (Table 10).

### Modulation of alternative splicing

The vast majority of human genes (>95%) are alternatively spliced by the spliceosome, which enables genes to express splice isoforms that often exhibit distinctive functions.^75,536^ Alternative splicing affects genes (such as *FGFR*, *ERG*, *VEGFA*, and *AR*) that are clearly linked to the etiology of PCa, allowing us to develop novel targeted therapies that modulate alternative splicing for the treatment of PCa. For example, alternative splicing of *FGFR2* leads to the expression of FGFR2(IIIb) and FGFR2(IIIc) isoforms, whereas the IIIb isoform is specific to epithelial cells, while the IIIc isoform is exclusively expressed in most mesenchymal cells. Therefore, this splicing switch is a sensor of the EMT that results in tumor growth and/or metastasis.^537^ Using the *FGFR2*-based splicing reporter as a readout, a recent preclinical study identified compounds such as nemadipine-A (a T-type calcium channel inhibitor), NNC-55-0396 dihydrochloride (a L-type Ca channel inhibitor), and naltrexone hydrochloride (an opioid antagonist) that change *FGFR2* splicing and induce an epithelial phenotype in PCa cells.^538^ Moreover, the *ERG* oncogene is fused with the *TMPRSS2* promoter in 50% of PCa cases, and the fusion most often occurs between the *ERG* exon 4 and exons 1 or 2 of *TMPRSS2*.^539^ Interestingly, a novel oligonucleotide-based agent that targets ERG by inducing skipping of its constitutive exon 4 resulted in a reduction in ERG protein levels and tumor growth in mice.^540^ Although these agents have not entered clinical practice yet, splicing events are a plausible mechanism for treating PCa, and further research is warranted.

## Future directions and conclusions

The arsenal of drugs available to treat advanced PCa has expanded significantly in recent years. Despite these advances, current treatment options have many limitations (Table 11), and more personalized treatment strategies and targeted agents are required for novel therapeutic options. In most cases, AR continues to be the primary molecular driver in CRPC patients, while effective therapies targeting AR are not always curative.^541^ AR overexpression, AR amplification, AR mutations, and AR variants (AR-Vs) are important mechanisms that contribute to drug resistance.^143,542,543^ Of note, drug resistance mediated by AR-Vs continues to be an issue, as it is very difficult to directly target AR-Vs.^541,544^ Despite the loss of AR-LBD, AR-Vs contain AR-DBD and are capable of transcriptional regulation. To date, AR-V7 is the type of AR-V most frequently detected in CRPC.^545,546^ AR-V7 was found to contribute to resistance to enzalutamide and abiraterone in PCa patients.^547^ In addition, other studies have demonstrated that other AR-Vs such as AR-V1, AR-V3, AR-V9, and ARv567es confer anti-AR drug resistance.^548,549^ Thus, further development of AR-V-targeted drugs such as AR-DBD or AR-NTD is required,^550,551^ since these domains are shared between full-length AR, LBD-mutant AR, and AR-Vs.

**Table 11:** Promises and limitations of therapeutic targets for PCa

Target	Promises	Limitations
AR signaling	• Backbone of systemic therapy • Multiple lines of treatment options available • Survival benefits	• Inevitability of castration resistance • Sexual dysfunction • Other complications such as increase fat mass
Bone microenvironment	• Prevention of skeletal-related events	• No survival benefits • Dental complications • Risk of hypocalcemia
PSMA	• High specificity • Diagnostic and therapeutic options available	• Issues of accessibility • PSMA PET might lead to inappropriate changes in PCa management or trial participation
DNA repair	• Promising response for selected patients	• Low incidence of DNA repair gene alterations in PCa • Unsubstantiated benefit for DNA repair gene alterations other than BRCA1/2 and ATM • Lack of evidence for OS benefit
Immune checkpoints	• Possibility for long-term cancer remission	• Generally poor response due to immunologically ‘cold’ tumor microenvironment • Eligible for very few PCa patients
Cell cycle	• Opportunity for synergistic success	• Limited clinical trials • Questionable safety profile
PI3K/AKT/mTOR signaling	• Opportunity for synergistic success	• Limited clinical trials • Biomarkers needed for patient selection
Epigenetic marks	• Mechanistically novel and promising	• Questionable safety profile • Limited efficacy
WNT signaling	• Mechanistically promising for late stage or refractory PCa	• Bone-related toxicity • Limited clinical trials

Remarkably, the number of patients with distant metastasis is rapidly increasing and shows no signs of cessation. Poor clinical outcomes are often observed following prolonged use of potent AR-targeted therapy, which is highly associated with treatment-induced neuroendocrine PCa (NEPC).^552^ Most NEPCs exhibit AR-indifference, drug resistance, and poor clinical outcomes^553^, as well as have high levels of genomic alterations, such as loss of function mutations of *PTEN, RB1*, and *TP53*.^554,555^ Moreover, other phenotypes, such as the double-negative (AR-null and NE-null) PCa or AR noncanonical function-dependent PCa also occur in patients,^508,556^ prompting investigators to examine oncogenic signaling pathways that drive these progressive and drug-resistant PCa. Several new pathways and key molecules have been identified, including GR, GATA2, IGF2, ONECUT2, POM121, AURKA, N-Myc, HP1α, PEG10, SRRM4, BRN2, SOX2, and PRMT5, which could be effective therapeutic targets for PCa.^365,557–566^

Notably, the pioneer factor FOXA1 mutation and overexpression could be an important factor in PCa.^406,567–573^ Gain-of-function mutations of *FOXA1* frequently occur in up to 9% and 13% of primary PCa and mCRPC cases, respectively.^569,573^ A previous study identified *FOXA1* single nucleotide variants in approximately 25% of the NEPC cases.^574^ Interestingly, a whole genome sequence study of PCa found that the rate of *FOXA1* mutation in Asian populations (~40%) is significantly higher than in western cohorts (~8%).^95^ Importantly, FOXA1 overexpression also suppresses the immune response, resulting in therapeutic resistance of ICIs in PCa.^348^ While it is well known that FOXA1 is very difficult to target, the proteolysis targeting chimeras (PROTACs), such as oligonucleotide-based PROTACs (O’PROTACs),^575^ present a new opportunity to develop novel anticancer agents targeting FOXA1 and its mutations.

More prospective clinical trials are urgently required for the optimization of treatment sequences and drug combinations that can delay resistance or decrease drug toxicities. Interestingly, a phase 2 study demonstrated that the use of abiraterone followed by enzalutamide led to a longer PSA progression-free interval compared with the reverse sequence.^576^ Results from a phase 1b trial that evaluated the combination of cabozantinib (a multityrosine kinase inhibitor) and atezolizumab (a PD-L1 inhibitor) in high-risk PCa are encouraging.^577^ Therefore, more prospective clinical studies regarding treatment sequences and drug combinations are required, including a combination of the new approved agent ^177^Lu-PSMA-617 either with PARP inhibitors or with AR signaling inhibitors, as well as with chemotherapy in the PSMA-positive population. Moreover, future studies should include a combination of EZH2 inhibitors with PARP inhibitors, tyrosine kinase inhibitors, CDK4/6 inhibitors, CDK7/9 inhibitors, AKT inhibitors, MEK inhibitors, CBP/p300 inhibitors, AURKA inhibitors, or chemotherapy for aggressive AR-negative PCa or NEPC.

PCa is an immune “cold” tumor that remains unresponsive to ICIs. Future directions also include the development of new immunotherapy agents or combinations that improve the efficacy of immunotherapy in PCa, including the combination of ICIs with inhibitors that target myeloid-derived suppressor cells (MDSC),^578,579^ cancer-associated fibroblasts (CAF),^580^ hypoxia,^581^ or other cancer immune response suppression factors such as KDM5B, PTPN2, SOCS1, ADAR1, MYC, and integrin αvβ6.^361,582–587^ PCa remains a complex and severe health issue globally, but technological advances, such as genomic sequencing and predictive algorithms, are constantly improving our understanding of the biology of this disease, thus facilitating the discovery of drugs with the best function and minimal toxicity. These advances are expected to promote precision medicine and improve clinical outcomes of PCa in the future.
